# Discovery and characterization of BRBV-sheep virus in nasal swabs from domestic sheep in China

**DOI:** 10.3389/fcimb.2024.1380708

**Published:** 2024-06-28

**Authors:** Yufei Zhang, Yang Li, Lemuge Qi, Tianyu Hang, Peng Wang, Yarong Wang, Caili Wu, Yongqin Wang, Xufen Wang, Lin Hou, Yaxing Ban, Zhidan Zhang, Weiguang Zhou

**Affiliations:** ^1^ College of Veterinary Medicine, Inner Mongolia Agricultural University, Hohhot, China; ^2^ Key Laboratory of Clinical Diagnosis and Treatment Technology in Animal Disease, Ministry of Agriculture, Hohhot, China; ^3^ The Spirit Jinyu Biological Pharmaceutical Co. Ltd., Hohhot, Inner Mongolia, China; ^4^ Animal Health and Slaughtering Management Stationin, Yulin, Shaanxi, China

**Keywords:** virome, metagenomic sequencing, bovine rhinitis B virus, complete genome, RT-qPCR, TaqMan probe, nucleotide deletion

## Abstract

**Introduction:**

The escalating occurrence of infectious disease outbreaks in humans and animals necessitates innovative, effective, and integrated research to better comprehend their transmission and dynamics. Viral infection in livestock has led to profound economic losses globally. Pneumonia is the prevalent cause of death in sheep. However, very few studies exist regarding virus-related pathogens in sheep. Metagenomics sequencing technologies in livestock research hold significant potential to elucidate these contingencies and enhance our understanding.

**Methods:**

Therefore, this study aims to characterize respiratory viromes in paired nasal swabs from Inner Mongolian feedlot sheep in China using metaviromic sequencing. Through deep sequencing, *de novo* assembly, and similarity searches using translated protein sequences, several previously uncharacterized and known viruses were identified in this study.

**Results:**

Among these discoveries, a novel Bovine Rhinitis B Virus (BRBV) (BRBV-sheep) strain was serendipitously detected in the nasal swabs of domestic sheep (Ovis aries). To facilitate further molecular epidemiological studies, the entire genome of BRBV-sheep was also determined. Owing to the unique sequence characteristics and phylogenetic position of BRBV-sheep, genetically distinct lineages of BRBV in sheep may exist. A TaqMan-based qRT-PCR assay targeting the 3D polymerase gene was developed and used to screen 592 clinical sheep specimens. The results showed that 44.59% of the samples (264/592) were positive. These findings suggest that BRBV sheep are widespread among Inner Mongolian herds.

**Conclusion:**

This discovery marks the initial identification of BRBV in sheep within Inner Mongolia, China. These findings contribute to our understanding of the epidemiology and genetic evolution of BRBV. Recognizing the presence of BRBV in sheep informs strategies for disease management and surveillance and the potential development of targeted interventions to control its spread.

## 1 Introduction

The Picornaviridae virus family is responsible for various diseases affecting humans and animals. This family encompasses 12 distinct genera: Enterovirus, Cardiovirus, Aphthovirus, Hepatovirus, Parechovirus, Erbovirus, Kobuvirus, Teschovirus, Sapelovirus, Senecavirus, Tremovirus, and Avihepatovirus. Picornaviruses are characterized by their genetic diversity and small, non-enveloped structures (Rai and Rieder, 2012; Lefkowitz et al., 2018). They contain a single-stranded positive-sense RNA genome, varying in length from 7–8.9 kilobases. Picornavirus classification hinges largely on their physiochemical properties, genome composition, and replication mechanisms.

Rhinoviruses were initially classified as a distinct genus within the Picornaviridae family. However, recent sequence and phylogenetic data analysis prompted the International Committee on Taxonomy of Viruses to reclassify the genus Rhinovirus. Human rhinoviruses are now placed within the genus Enterovirus, while bovine rhinoviruses are reclassified under the genus Aphthovirus (Hollister et al., 2008; Lefkowitz et al., 2018). Bovine rhinitis viruses (BRV) comprise two species: bovine rhinitis A viruses (BRAV-1 and BRAV-2) and bovine rhinitis B virus (BRBV). The latter species has a single serotype, referred to as bovine rhinitis B virus type-1 (BRBV-1). Reed obtained the first BRBV EC-11 isolate in England in 1964 from the lungs of a respiratory-diseased pathogen-free calf (Bogel, 1966).

The genomic RNA of BRBV encodes a large polyprotein, broken down by viral proteases into structural (capsid) and non-structural polypeptides. Investigating the pathogenesis of BRBV involves intranasal inoculation in calves (Reed et al., 1971; Cunningham et al., 2012). Clinical manifestations include fever, nasal discharge, and elevated respiratory rate. Histological analysis of the turbinates and trachea revealed epithelial necrosis, while the lungs exhibited signs of interstitial pneumonia. However, the precise pathogenic mechanisms of BRBV remain largely unclear.

The continuous advancement of high-throughput sequencing technologies has substantially led to reduced costs associated with DNA sequencing, expanding access to this technology for a broader population. In recent decades, the intrusion into animal and livestock reservoirs has resulted in profoundly impactful epidemics (Cunningham et al., 2012; Suminda et al., 2022). Livestock diseases result in direct and indirect economic losses, exerting substantial social and environmental impacts (Tomley and Shirley, 2009; Suminda et al., 2022). Pneumonia stands as the most common cause of death in sheep. While Pasteurella species are frequently linked to pneumonia, other agents also play a role (Cunningham et al., 2012). However, very few reports on virus-related pathogens in sheep exist. Employing high-throughput sequencing technology is significantly important for identifying respiratory pathogenic microorganisms. Therefore, this study aims to characterize respiratory viromes in paired nasal swabs collected from Inner Mongolian feedlot sheep in China using metaviromic sequencing.

This study identified a novel strain of BRBV in nasal swab samples collected from sheep, showing prevalence among sheep in Inner Mongolia, China. Furthermore, we developed a rapid and reliable real-time reverse transcription-quantitative polymerase chain reaction (RT-qPCR) assay, utilizing TaqMan probes to enhance our comprehension of BRBV-sheep epidemiology.

## 2 Materials and methods

### 2.1 Sample collection

Daily visual examinations and assessments of sheep for clinical presentations were performed by experienced feedlot personnel. Sheep with symptoms such as depression, respiratory distress, coughing, and nasal and ocular discharge underwent further examinations at a feedlot hospital facility. This study included thirty sheep with symptoms such as depression, respiratory distress, cough, and nasal and ocular discharge. To collect samples, long swabs (15 cm) equipped with a guarded rayon bud were inserted into the nostrils, wiped with a paper towel, and then advanced into the nasopharynx. The samples were collected from the nasopharynx by extending the swab beyond the guard and vigorously agitating it back and forth against the mucosal surface. By retracting the swab behind the guard, the complete deep nasal swab was withdrawn from the nasal passageway. The swab tip was placed into a transport tube containing liquid Amies transport medium. Five swab tips were randomly divided into 6 experimental groups. It was then separated from the rest of the swab using scissors. The samples were immediately stored in a polystyrene cooler with ice and transported to Inner Mongolia Agricultural University in Hohhot. The protocol for the animal research was reviewed and approved by the Institutional Animal Care and Use Committee at Inner Mongolia Agricultural University, China.

### 2.2 Sample preparation

The swabs washes underwent centrifugation at 13,000 × g for 5 min. A subsample of the supernatant from each sample underwent incubation for 90 min at 37°C with 20 µL of TURBO DNase buffer, 24 units of DNase (Life Technologies, Beijing, China), and 20 units of RNase ONE Ribonuclease (Promega, Beijing, China) to eliminate host nucleic acids. Subsequently, viral nucleic acids were extracted using a viral nucleic acid purification kit (QIAamp MinElute virus spin kit, Qiagen, Shanghai, China) per the manufacturer’s instructions and eluted with 30 µL of nuclease-free water. Utilizing a Superscript III reverse transcription kit (Roche, Shanghai, China), reverse transcription was performed with primers comprising a known 20 nt sequence followed by (N)6 at the 3’ end. Sequenase 2.0 (Affymetrix, Ohio, USA) was used for second-strand synthesis.

### 2.3 Metagenomic sequencing

Double-stranded cDNA was purified using a Qiagen Minelute PCR spin column. Subsequently, it was amplified with primers identical to the known 20 bp region of the random hexamer-containing primer. The resultant amplicons were purified on a Qiagen MinElute PCR spin column. They were then quantified using a Qubit fluorometer (Life Technologies, USA). Following that, the samples were diluted to a concentration of 0.2 ng/µL, and 5 µL was used for sequencing library preparation employing the Nextera XT library preparation kit (Illumina, USA), according to the manufacturer’s instructions. The pooled barcoded libraries were subsequently sequenced on an Illumina Novaseq6000 PE150 instrument using paired 150 bp reads.

### 2.4 Bioinformatic analysis

The demultiplexed raw data underwent quality trimming with Trimmomatic‐0.32 (Bolger et al., 2014), with the following parameters: a minimum length and Phred score of 50 and 20 (Zhang et al., 2019), respectively. The quality-trimmed reads were subsequently aligned to the Ovis aries reference genome using Bowtie2 (Li et al., 2009; Zhang et al., 2019; Wang et al., 2023). Samtools (Li et al., 2009; Wang et al., 2023) was used to identify any unmapped reads. Following that, unmapped reads were extracted from the original FASTQ files using cdbyank. Using default parameters, Trinity (Celaj et al., 2014) was then used to perform *de novo* assembly of unmapped reads for each sample. The assembled contigs were subsequently aligned to the viral Sequence (RefSeq) database (Brister et al., 2015) using BLASTn (V2.10.0 +). To detect virus-like sequences, contigs that produced alignments of at least 100 base pairs in length with expectation (e) values <10^−3^ were further analyzed. The viral reads in each sample library were quantified by mapping reads from each sample onto assembled contig sequences using Bowtie2. The original data files were deposited in the National Center for Biotechnology Information (NCBI) Sequence Read Database under the accession number PRJNA1040299.

To compute sequence identities at the nucleotide (nt) and amino acid (aa) levels, the MegAlign program was employed. Subsequently, various evolutionary models were assessed, and the most suitable model was employed to construct phylogenetic trees using Mega7 software. To ensure the reliability of the tree topologies, 1000 bootstrap resampling replicates were conducted. RDP 4.101 was employed to conduct recombination analyses, employing multiple algorithms, including RDP, GeneConv, Chimaera, MaxChi, BootScan, SiScan, 3Seq, and Phylpro. Recombination events supported by a minimum of five algorithms were deemed reliable (Zhou et al., 2023). To identify the natural selection processes influencing the codons of the BRBV-sheep ORFs, selection pressure was assessed. The following algorithms were used for the selection analysis in DataMonkey [https://www.datamonkey.org/ (accessed on January 12, 2020)]: Fixed Effects Likelihood (FEL), Fast Unconstrained Bayesian Approximation (FUBAR), Single-Likelihood Ancestor Counting (SLAC), and Mixed Effects Model of Evolution (MEME) (Rai et al., 2015). FUBAR’s p-value was 0.9, whereas MEME, FEL, and SLAC’s were 0.1 and 0.9, respectively. Assuming that each site’s selection pressure remained constant across the phylogeny, the definitions of positive, neutral, and negative selection were, respectively, dN/dS > 1, dN/dS = 1, and dN/dS < 1.

### 2.5 Primer and probe design for BRBV-Sheep

Full-length sequences of BRBV-Sheep were retrieved from the GenBank database (https://www.ncbi.nlm.nih.gov/genbank/) (Accession Number: MT160419). A pair of specific primers and probes was designed using Primer 5.0 software. This design was guided based on DNAMAN comparison and complied with the RT-qPCR primer design criteria, targeting highly conserved regions in the virus sequences. Primer specificity was confirmed through primer-blast comparisons using the NCBI database. Following this confirmation, specific primers capable of producing amplicons were screened via RT-PCR and subsequent analysis with agarose gel electrophoresis. **Table 1** lists the primers (BRBV-169-F/R) and probe (BRBV-169-Probe) used in this study. The amplicon size was 169 bp.

**Table 1:** Selection pressure analysis of BRBV-sheep strains collected in China.

ANALYSIS	NEGATIVE SITES				POSITIVE SITES			
	NO.	CODON SITE	AVG. dN/dS	P-VALUE	NO.	CODON SITE	AVG. dN/dS	P-VALUE
SLAC	626	N.A.	0.482	0.0096–0.05	6	13,33,406,1426,1686,1982	4.140	0.0168–0.05
FEL	64	N.A.	N.A.	0.0214–0.05	10	12,92,434,708,1392,1559,1793,1827,2131,2143	Infinite	0.0078–0.05
FUBAR	2154	N.A.	N.A.	N.A.	1	406	N.A.	0.017
MEME	N.A.	N.A.	N.A.	N.A.	44	36,61,130,163,299,305,308,347,352,353,381,383,385,406,433,434,479,499,528,572,573,580,599,600,626,643,661,686,714,739,772,776,789,837,844,874,880,937,1162,1469,1530,2184,2200,2204	N.A.	0.00001–0.05

### 2.6 Preparation of positive control plasmid

A BRBV-Sheep isolate (Accession Number: MT160419) present in the laboratory was selected to create a positive control plasmid. A portion of the genome was amplified using the BRBV-169-F and BRBV-169-R primers via RT-PCR. The PCR products obtained were sent to Sangon Biotech (Shanghai, China) for sequencing, which confirmed a 100% match with the target sequence. Subsequently, the PCR products were purified using a DNA Gel Extraction Kit (Takara, Dalian, China). Purified DNA fragments were obtained and then cloned into the pMD19-T vector (Takara, Dalian, China) to generate a plasmid named pMD19T-BRBV-Sheep, serving as a positive control and template. The plasmid underwent sequencing to verify its accuracy and establish a standard curve. The plasmid concentration was determined via absorbance measurements, and the copy number was calculated using a previously described method (Lefkowitz et al., 2018; Xie et al., 2021). The plasmid pMD19T-BRBV-Sheep, serving as the standard, was diluted to a concentration of 10^9^ copies per assay and then homogenized at an equal concentration. The resulting plasmid mixture was diluted to achieve a concentration range, spanning from 10^9^ copies per assay to 1 copy per assay.

### 2.7 Optimization of reverse transcriptase quantitative polymerase chain reaction for bovine rhinitis B virus

The QIAamp Viral RNA Mini Kit (Qiagen, Shanghai, China) was used to extract genomic RNA, followed by reverse transcription to cDNA using a Reverse Transcriptase AMV kit (Roche, Shanghai, China). Conditions for RT-qPCR were optimized in triplicate using PerfectStart^®^ II Probe qPCR SuperMix (TransGen Biotech Co., Beijing, China). The ABI7500 FAST fluorescent qPCR system (Applied Biosystems, Foster City, CA, USA) was employed to conduct the PCR. The reaction mixture was prepared following the instructions provided in the kit, and the conditions were adjusted accordingly. The initial reaction mixture, measuring 25 μL, included 0.5 μL of Passive Reference Dye II (50 ×), 12.5 μL of 2 × PerfectStart^®^ II Probe qPCR SuperMix, 1.0 μL of BRBV-169-F, 1.0 μL of BRBV-169-R, 1.0 μL of BRBV-144-Probe, 2.0 μL of template cDNA, and 7 μL of RNase-free dH_2_O. The initial reaction program involved 45 cycles of 98°C for 10 s, followed by 60°C for 30 s, with fluorescence signal scanning conducted at the final stage of each cycle.

To optimize the efficacy of the assay, the optimal fluorescence and threshold cycle values were assessed. This assessment involved varying the reaction temperature (50–60°C) and concentrations of primers and probes (0.1 μmol/L, 0.2 μmol/L, 0.4 μmol/L, and 0.5 μmol/L) in a 25 μL total system. The template concentration was maintained at 1 μmol/L, and the thermal cycle condition included an initial step of 95°C for 30 s, followed by 95°C for 5 s, 60°C for 30 s, and 45 cycles. Using 10-fold dilutions of the recombinant plasmid (10^8^–10^1^ copies/μL) and the optimized system and conditions, a standard curve was generated. The optimization tests were performed in triplicate, maintaining the threshold at the default setting. The optimal reaction parameters were established based on the cycle threshold (C_T_) value and fluorescence intensity. In this study, sensitivity, quantification, specificity, reproducibility, and sample tests were conducted under the optimized reaction conditions.

### 2.8 Sensitivity test

After optimizing the RT-qPCR reaction, sensitivity was assessed utilizing a continuously diluted recombinant plasmid containing BRBV-Sheep cDNA as a template. The dilutions ranged from 1 ×10^8^ to 1 ×10^1^ copies/μL to confirm the detection limit. The results were then compared to those obtained using the previously developed RT-PCR. The detection threshold was established as the minimum pMD19-BRBV-Sheep copy count that produced at least one positive result per replicate. A positive result was confirmed when a reliable C_T_ value was obtained. This was determined based on the presence of a sigmoidal curve in the plot of absolute fluorescence values aligned with the amplification kinetics (Xie et al., 2021).

### 2.9 Specificity and reproducibility tests

The specificity of RT-qPCR was assessed using seven pathogens: Bovine Rhinotracheitis Virus (IBRV), bovine parainfluenza virus type 3 (BPIV-3), Pasteurella multocida serotype A, Mannheimia haemolytica, Pasteurella multocida serotype B, Mycoplasma bovis, and Klebsiella pneumoniae. These viruses were subjected to DNA or RNA dilution. BRBV sheep RNA and ddH_2_O were positive and negative controls, respectively, ensuring equal concentrations.

To assess the intra- and inter-batch reproducibility of the RT-qPCR method, pMD19-BRBV-Sheep templates at concentrations ranging from 1×10^6^ to 1×10^1^ copies/μL were utilized. Each sample was tested in triplicate within the same plate, and the assay was conducted thrice across different plates and at varying intervals. The mean Cq, standard deviation (SD), and coefficient of variation (CV) were calculated and analyzed based on the Cq values of each replicate.

### 2.10 Test with clinical samples

**Figure 1:**
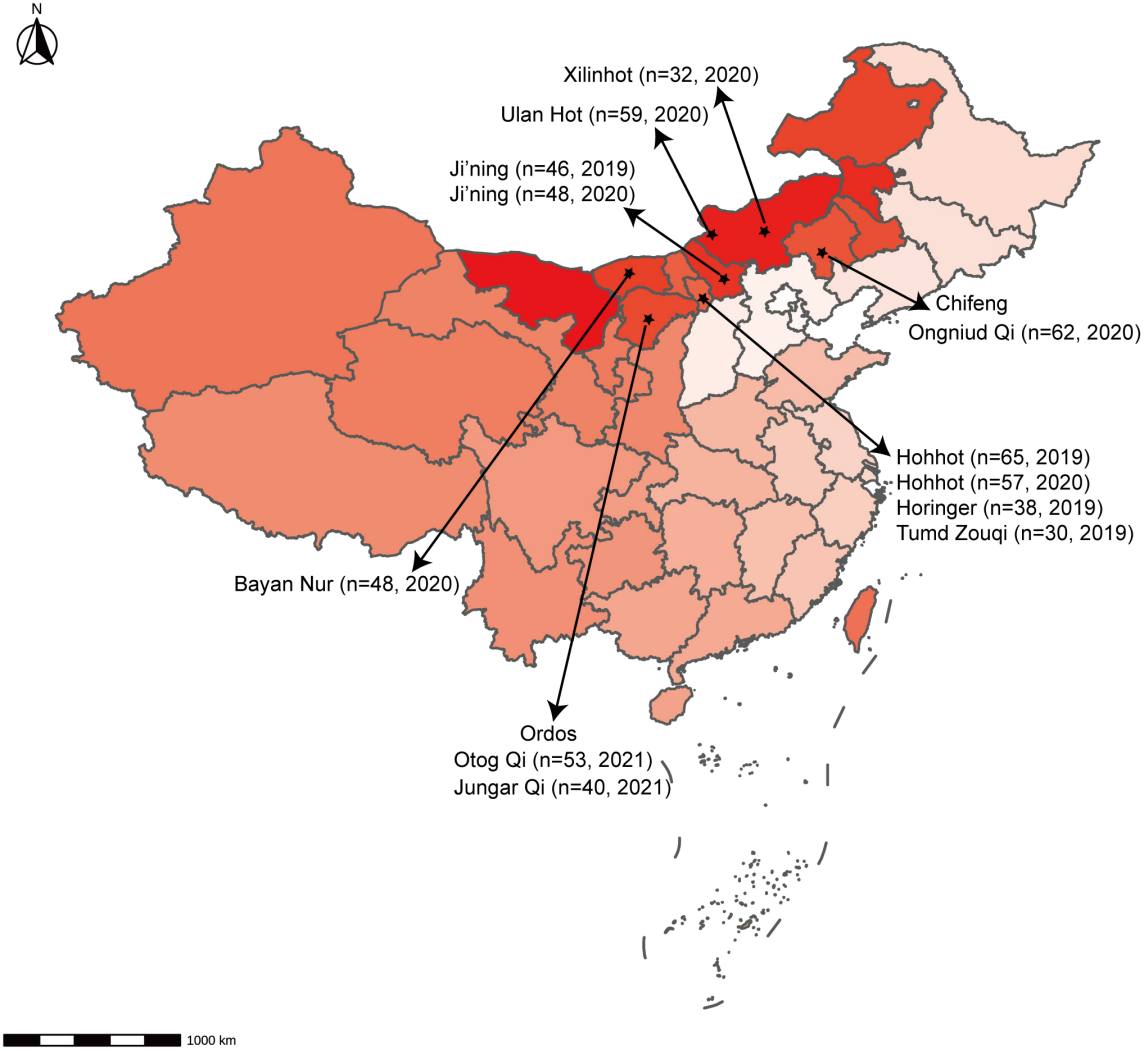
Geographical distribution of samples. The number of animals and the time of sampling are indicated.

Overall, 592 nasopharyngeal swab samples from sheep in the Inner Mongolia Autonomous Region, China, were collected from various locations: Hohhot, Ji’ning, Bayan Nur, Ulan Hot, Ordos, Chifeng, and Xilinhot (**Figure 1**). These samples were then tested using the newly developed RT-qPCR assay. RT-qPCR and RT-PCR were used for detection, with sequencing employed to detect false-positive reactions. The positivity rate was calculated for each animal within each city.

### 2.11 Statistical analysis

The chi-square test was used to examine the detection outcomes of 592 suspected sheep nasopharyngeal swab samples obtained via RT-qPCR and RT-PCR, and the detection rate and accuracy were subsequently compared. The concordance between assays was assessed using Cohen’s kappa statistic, with a 95% confidence interval (CI). Following the detection of inconsistent results in 592 sheep nasopharyngeal swab samples, the viral gene of interest was amplified using RNA obtained from those samples. Subsequently, it was subjected to Sanger sequencing performed by a commercial sequencing enterprise, Sangon Biotech (Shanghai, China).

## 3 Results

### 3.1 Diverse viruses found in sheep nasal cavity

**Figure 2:**
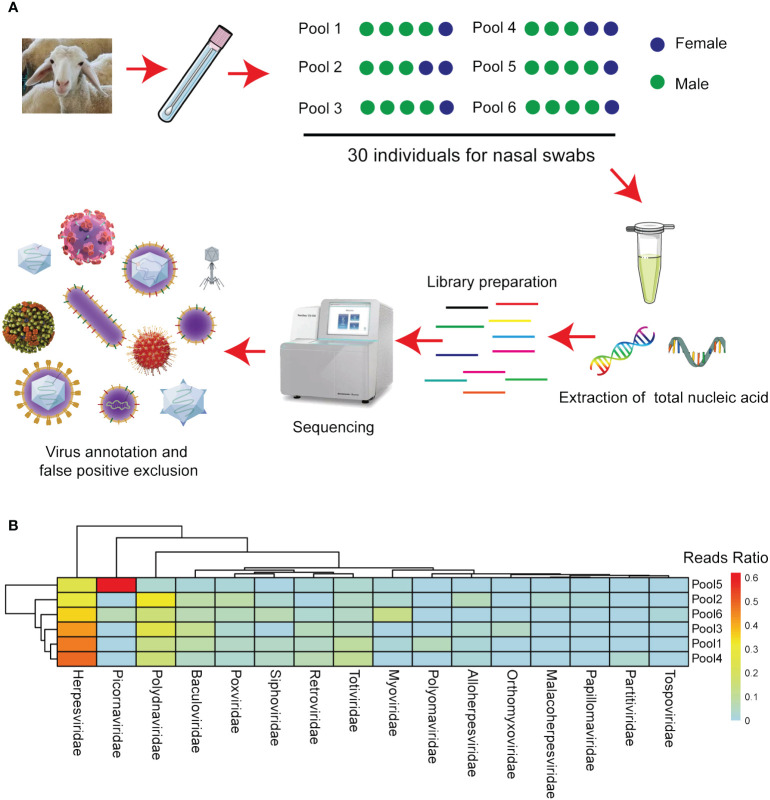
Next-generation sequencing identified the viruses in the various sheep sample pools. **(A)** Flow diagram depicting the sampling and next-generation sequencing for the virome. **(B)** The heatmap illustrates the distribution of viral reads across taxonomic ranks (family level).

To explore potential viral agents in sheep, 30 nasal swabs were collected from sheep in Inner Mongolia, Northern China, exhibiting respiratory symptoms following long-distance transportation. The five nasal swabs were pooled, resulting in six samples. Nucleic acids were then extracted from these samples, utilized to create nucleic acid libraries, and sequenced using the Illumina MiSeq platform (**Figure 2A**). From the sequencing results, 59409196410 bases were obtained from the six samples, constituting 408974828 reads. All raw sequences are accessible in the NCBI database (Bioproject ID: PRJNA1040299). After filtering out other irrelevant nucleic acids from the original sample, 1746168 viral sequences were obtained. The clean reads obtained were subjected to *de novo* assembly, revealing that phage, plant virus, invertebrate virus, human virus, bovine virus, and sheep virus. These sequences accounted for 42%, 11%, 1.5%, 28.5%, 4.3%, 4.1%, respectively. Other vertebrate, marine, and unclassified viruses accounted for 5.4%, 0.7%, and 2.5%, respectively. These viruses were categorized into the orders Alloherpesviridae, Baculoviridae, Herpesviridae, Malacoherpesviridae, Myoviridae, Orthomyxoviridae, Papillomaviridae, Partitiviridae, Picornaviridae, Polydnaviridae, Polyomaviridae, Poxviridae, Retroviridae, Siphoviridae, Tospoviridae, and Totiviridae based on their sequence similarity, genome structure, and phylogenetic relationships (**Figure 2B**).

### 3.2 Genomic characterization of BRBV-sheep

**Figure 3:**
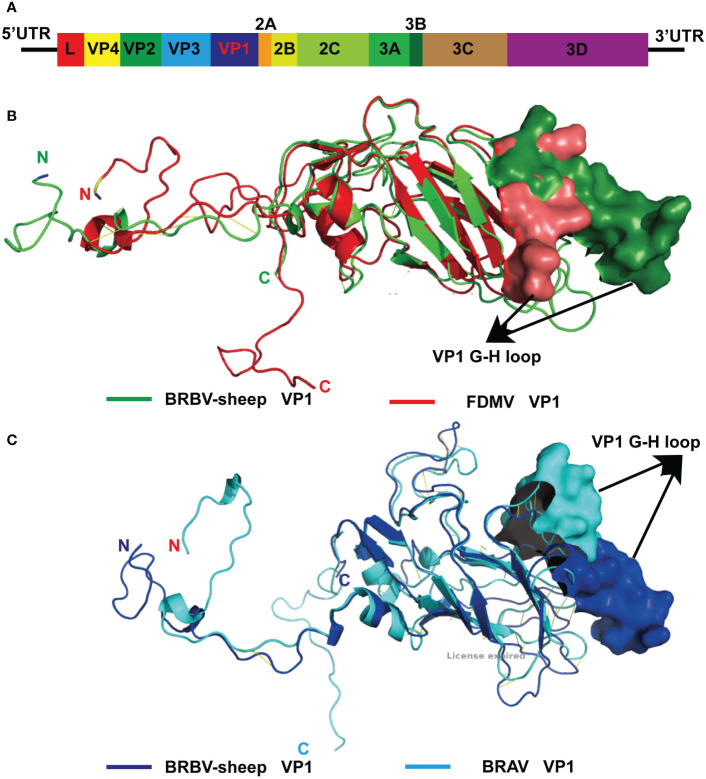
Modeled structure comparison of VP1 structural proteins from BRBV-sheep and FMDV or BRAV. **(A)**Genome organization of the BRBV-sheep identified in this study. **(B)** Modeled structures of VP1 structural proteins from BRBV-sheep (Green) and FMDV (Red). N- and C-terminus of the proteins in the structure are marked. **(C)** Modeled structures of VP1 structural proteins from BRBV-sheep (Blue) and FMDV (Cyan). N- and C-terminus of the proteins in the structure are marked.

From the metagenomics sequencing data, nucleic acids annotated to BRBV were identified in four nasal swab sample pools, and they were successfully spliced into the whole genome sequence of BRBV-sheep. The full-length genome of BRBV-sheep, spanning 7,578 nucleotides, extended from a putative poly(C) tract at the 5′ UTR end to the poly(A) tail at the 3′ end. The BRBV-sheep genome length spanned 7,578 nucleotides, showing a G+C content of 47.12%. This included an untranslated region of 726 nucleotides and a coding region of 6,852 nucleotides encoding 2,284 amino acids. The genome of BRBV-sheep encodes eight nonstructural proteins, including leaders 2A, 2 B, 2C, 3A, 3 B, 3C, and 3D, and four structural proteins: VP1, VP2, VP3, and VP4 (**Figure 3A**). The whole genome sequence has been deposited in the NCBI database (OQ547742.1).

#### 3.2.1 5′ UTR

The 5′ UTR poly(C) of BRBV-sheep exhibited nucleotide sequence identities of 91.1%, 51.9%, 34.2%, and 32.1% with BRBV (KY432299.1), BRAV (NC_038303.1), FMDV (AJ133359.1), and ERAV (NC_039209.1), respectively.

#### 3.2.2 L^pro^

ORF scan analysis indicated variability in Lpro of BRBV-sheep Lpro compared to other Aphthoviruses, such as FMDV and BRAV. The Lpro active site (highlighted in yellow) and translation initiation factor-binding sites (highlighted in cyan) remained conserved (**Supplementary Figures S1**–**S2**-**5**). Moreover, these sites were shared by the BRBV sheep with other members of the Aphthovirus genus (**Supplementary Figures 1**–**S2**-**5**).

#### 3.2.3 Structural proteins

The structural protein VP4—the first protein encoded by the structural protein region—exhibits the highest degree of conservation among Aphthoviruses and BRVs. However, BRVs featured a longer VP4 protein than other aphthoviruses. The remaining portion of the VP2 protein and all of the VP3 and VP1 proteins of the BRVs (BRBV-sheep, BRBVs, and BRAVs) exhibited distinct differences from those observed in FMDV. Furthermore, the distinctive RGD tripeptide—acting as a cell surface binding site in the VP1 G-H loop of FMDV—was notably absent in all other aphthoviruses (**Supplementary Figure 1**, **Supplementary Figures 2**-**5**). This finding suggests an alternative mechanism for their interaction and attachment to the cell surface. Given the absence of the RGD tripeptide in BRBV-sheep VP1, structural models of FMDV and BRBV-sheep were generated and compared. When overlaid, the structural models of the two proteins showed complete disorder in the G-H loop region of the BRBV-sheep VP1 molecule. This observation further bolsters the hypothesis of an alternative virus-receptor interaction occurring on the cell surface (**Figure 3B**). Furthermore, when the structural models of BRBV sheep VP1 and BRAV VP1 were prepared and compared, the G-H loop region was observed to partially overlap only. This observation further reinforces the possibility of utilizing distinct cell surface receptors (**Figure 3C**).

#### 3.2.4 Non-structural proteins

All Aphthoviruses and many other Picornaviruses encode the same C-terminal ribosome-skipping motif NPG↓P as 2A. The N-terminus exhibited more variability than the rest of the protein, suggesting potentially reduced functionality in this region. Clear differences were observed between the BRVs, encompassing the BRBV-sheep reported in this study and FMDV (**Supplementary Figure 1**, **Supplementary Figures 2**-**5**), particularly in the sizes of the 2 B and 3A proteins and the count of encoded 3 B peptides. Compared with FMDV, BRAV and other Aphthoviruses exhibited a conserved 2C-ATPase. FMDV encoded two copies of nonstructural protein 3 B, while BRBV-sheep and other Aphthoviruses encoded only one copy. Finally, 3Cpro and 3Dpol (highlighted in yellow) contain the most conserved and catalytically important residues of the two proteins, and these residues are conserved in all viruses compared in this study.

### 3.3 Phylogenetic analysis of BRBV-sheep

**Figure 4:**
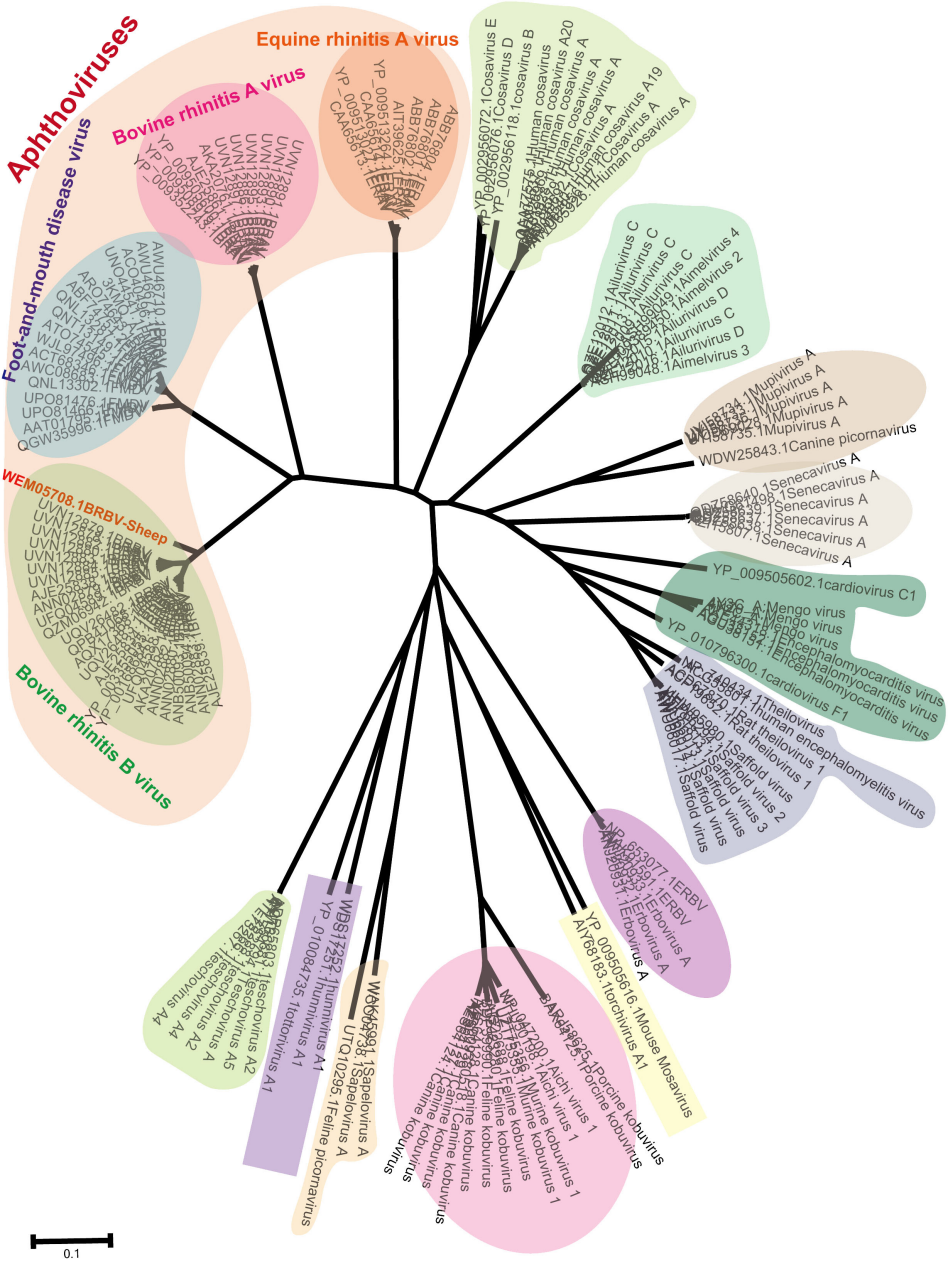
Phylogenetic tree showing BRBV-sheep based on the viral 3Dpol non-structural protein. Neighbor-joining phylogenetic trees were built using the JTT model for amino acid substitutions (pairwise deletions only) for 3Dpol non-structural proteins encompassing BRBV sheep and all known members within Picornaviridae.

Phylogenetic trees were constructed using neighbor-joining methods based on the most conserved non-structural protein, 3D^pol^. The BRBV-sheep sequence reported in this study and a set of classified Picornaviruses were used to elucidate the evolutionary history of BRBV-sheep in Picornaviridae. The aphthoviruses exhibited a similar clustering in the NJ-JTT phylogenetic tree of the 3D^pol^ region (**Figure 4**). Additionally, BRBV-sheep constituted a distinct branch in the phylogenetic analysis (**Figure 4**), indicating its unique evolutionary trajectory. Other aphthovirus genera, including FMDV, BRAV, and ERAV, formed separate nodes. However, all aphthoviruses were clustered within the Aphthovirus genus, suggesting their close evolutionary relationships.

### 3.4 Evolutionary history of BRBV-sheep

Positive selection represents a significant mechanism in RNA virus evolution. Initially, we examined the selection pressure among aphthoviruses, concentrating on the closest relatives to BRBV—specifically, the BRBV-sheep strain, BRBV, FMDV, and BRAV. To accomplish this, we utilized various methodologies accessible on the Datamonkey web server. These methodologies encompass single likelihood ancestor counting (SLAC) analysis, fixed effect likelihood (FEL), and fast-unconstrained Bayesian AppRoximation (FUBAR) methods. They aimed to detect evolutionary evidence of positive selection by estimating global ω values. Two positively selected codons, specifically 406 and 434, were detected using at least two methods. Site 406 was identified as positively selected using three methods (**Table 1**). The three methods effectively identified numerous negative selection sites in BRVs, with SLAC, FEL, and FUBAR detecting 626, 64, and 2154 sites, respectively. The FEL method was used to effectively detect 10 codon sites (12, 92, 434, 708, 1392, 1559, 1793, 1827, 2131, and 2143) in the structural and non-structural protein regions under positive selection. FUBAR detected a single codon site exhibiting positive selection among BRBVs. Furthermore, the MEME method identified 44 sites that underwent positive purifying selection among BRBVs, evenly distributed across the ORF. This finding strongly suggests robust positive selection within the BRBV species.

**Figure 5:**
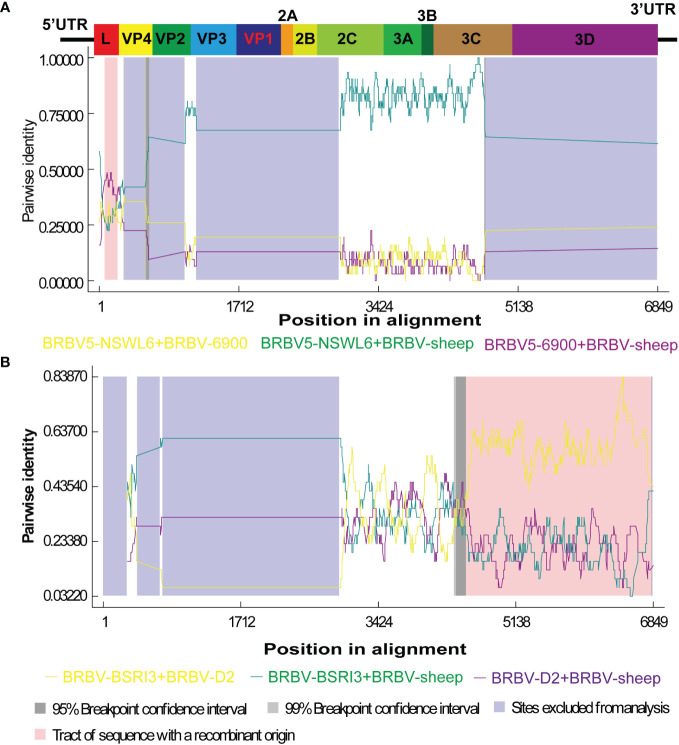
Recombination analysis of BRBV-sheep. **(A)** BRBV-NSWL6 (GenBank Accession: OP020154.1) and BRBV-6900 (GenBank Accession: MZ574106.1) are identified as the putative major and minor parental strains, respectively. **(B)** BRBV-BSRI3 (GenBank Accession: KP264975.1) and BRBV-D2 (GenBank Accession: OL410605.1) are considered the major and minor parents, respectively.

Recombination is believed to play a significant role in RNA virus evolution. The most common first step in assessing the probability of recombination events in a set of nucleotide sequences involves searching for recombination breakpoints. The BRBV sequences underwent recombination analysis using RDP 4.101. Nevertheless, demonstrating a recombination event as significant should involve using multiple methods. We deemed an event significant only when it is supported based on the application of three or more methods. Thus, we focused on the results generated by three of the nine algorithms (RDP, GENECONV, CHIMAERA, MAXCHI, BOOTSCAN, PHYLPRO, LARD, SISCAN, and 3SEQ). A breakpoint was identified across the whole genome, ranging from nt 5 to nt 253, with the recombinant region located in the Lpro gene (**Figure 5A**). BRBV-NSWL6 (GenBank Accession: OP020154.1) and BRBV-6900 (GenBank Accession: MZ574106.1) were identified as the putative major and minor parental strains, respectively. Furthermore, the recombinant region is depicted in the 3C and 3D genes, spanning from nt 4372 to nt 6840 in the entire genome (**Figure 5B**). Among the parental strains, BRBV-BSRI3 (GenBank Accession: KP264975.1) and BRBV-D2 (GenBank Accession: OL410605.1) were thought to be the major and minor parents, respectively.

### 3.5 Optimized conditions for BRBV-sheep reverse transcriptase quantitative polymerase chain reaction

**Figure 6:**
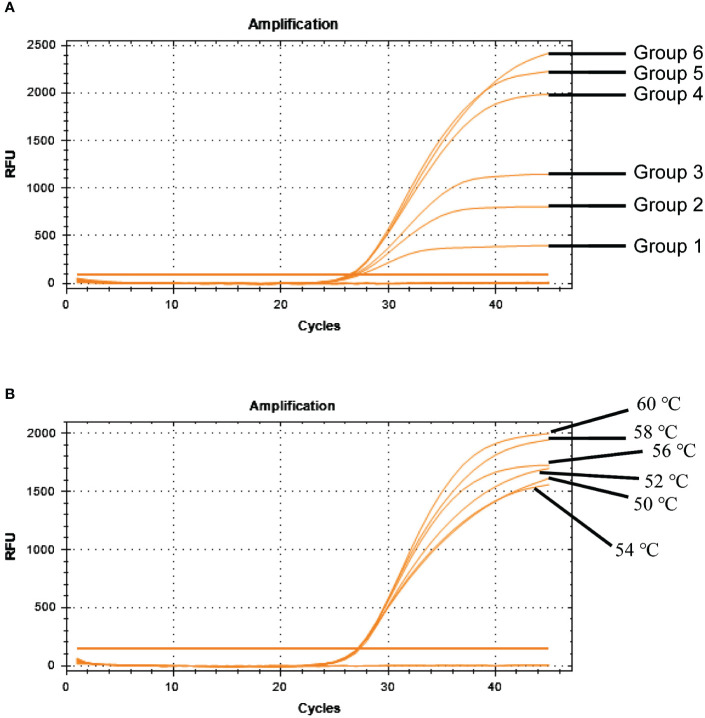
Optimization of BRBV-sheep qRT-PCR reaction conditions. **(A)** Optimization of probe and primer concentrations. **Table 2** shows probe and primer concentrations. **(B)** Optimization of annealing temperatures.

TaqMan-based qRT-PCR was optimized by identifying optimal primer and probe concentrations. qRT-PCR was performed on 1×10^6^ copies/μL of a positive control plasmid (**Table 2**). **Figure 6A** shows the results. The fourth group exhibited the most favorable fluorescence value, CT value, and amplification curve among the six groups. Hence, the optimal primer and probe concentrations were 1.00 μL (200 nmol) and 1.00 μL (200 nmol), respectively. Six temperature gradients (60.0°C, 58.0°C, 56.0°C, 54.0°C, 52.0°C, and 50.0°C) were established to conduct qRT-PCR on 1×10^6^ copies/μL of the positive control plasmid. **Figure 6B** shows the results. At the annealing temperature of 60°C, the fluorescence value reached its peak. Consequently, 60°C was determined as the optimal annealing temperature for this method.

**Table 2:** Optimization results of BRBV-sheep qRT-PCR reaction conditions.

Reaction conditions	Concentrations of annealing temperature		*Ct* value			Mean *Ct* value
**Probe and Primer concentrations**	Group 1	Probe(100 nmol/L) Primer(100 nmol/L)	27.39	27.38	27.40	27.39
	Group 2	Probe(100 nmol/L) Primer(150 nmol/L)	27.10	27.06	27.05	27.07
	Group 3	Probe(200 nmol/L) Primer(150 nmol/L)	26.90	26.85	26.75	26.80
	Group 4	Probe(200 nmol/L) Primer(200 nmol/L)	26.15	26.35	26.40	26.30
	Group 5	Probe(300 nmol/L) Primer(300 nmol/L)	26.58	26.30	26.44	26.44
	Group 6	Probe(400 nmol/L) Primer(500 nmol/L)	26.65	26.50	26.65	26.60
**Annealing temperature**	50°C		26.75	26.85	26.95	26.85
	52°C		26.43	26.89	26.93	26.75
	54°C		26.45	26.81	26.90	26.72
	56°C		26.51	26.82	26.83	26.74
	58°C		27.32	27.00	26.74	27.02
	60°C		26.41	26.70	26.78	26.63

### 3.6 Sensitivity assessment and limit of detection using TaqMan-based quantitative real-time RT-PCR for BRBV-sheep

**Figure 7:**
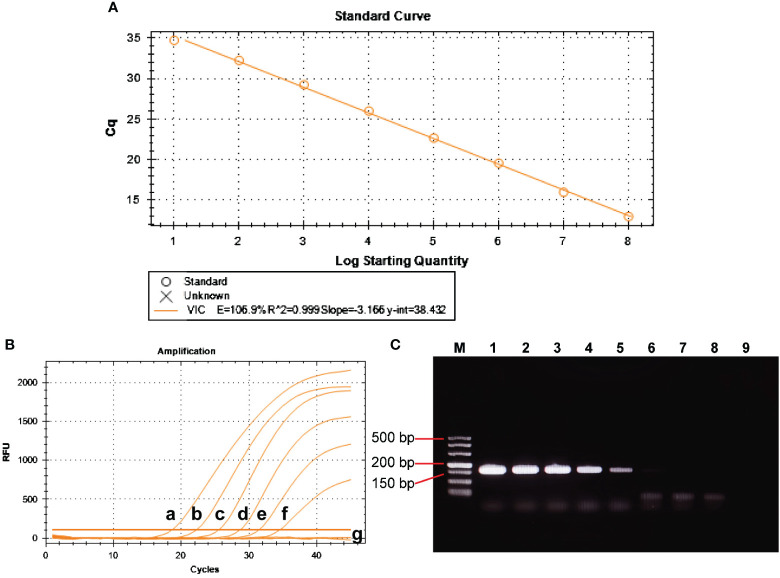
Analytical sensitivity of BRBV-sheep qRT-PCR assays. **(A)** Standard curve for the qRT-PCR assay of BRBV-sheep. A 10-fold serial dilution, ranging from 1 × 10^8^ to 1 × 10^1^ copies/μL of RNA standard, was assessed using the qRT-PCR. Three replicates were examined for each dilution. **(B)** Sensitivity tests for the qRT-PCR assay of BRBV-sheep. 10-fold serial dilutions of the RNA standard were used to perform the qRT-PCR to obtain the standard curve of the assay. The lowest detectable copy number was up to 10 copies/μL. **(C)** Sensitivity tests for conventional RT-PCR of BRBV-sheep. Tracks 1 to 8 represent the templates, with concentrations ranging from 1 × 10^7^ to 1 × 10^0^ copies/μL. Track 9 denotes negative control. The lowest detectable copy number was up to 1.0 × 10^3^ copies/μL.

The positive control plasmid was diluted at 10-fold multiplicity and used as a template, ranging from 1×10^8^ to 1×10^1^ copies/μL to obtain the standard curve for BRBV-sheep qRT-PCR. **Figure 7A** shows the results, indicating a coefficient of determination (R^2^) of 0.999 and PCR efficiency of 106.9%. These findings suggest a strong linear relationship in the standard curve, indicating the high reliability of the established method.

Amplification was performed using 1×10^6^ to 1×10^1^ copies/µL of positive control plasmid as a template. **Figures 7B, C** show the results. The sensitivity of qRT-PCR was 1.0×10^1^ copies/µL, while the lowest detection amount of RT-PCR was 1.0×10^3^ copies/µL. This finding suggests the method developed in this study exhibited sensitivity. The limit of detection (LOD) for the qRT-PCR assay was determined using tenfold dilutions of the BRBV-sheep positive control plasmid, encompassing 1×10^6^ to 1×10^1^ copies/μL. The LOD for the positive control plasmid using qRT-PCR was 100 copies/μL. However, the inter-assay qRT-PCR did not demonstrate repeatability in the standard curves for 1×10^1^ duplicate dilutions of the positive control plasmid. No fluorescence changes were observed in the negative control group, suggesting that the BRBV-sheep assay exhibited a detection limit of approximately 10 copies.

### 3.7 Specificity of TaqMan-based quantitative real-time RT-PCR for BRBV sheep

**Figure 8:**
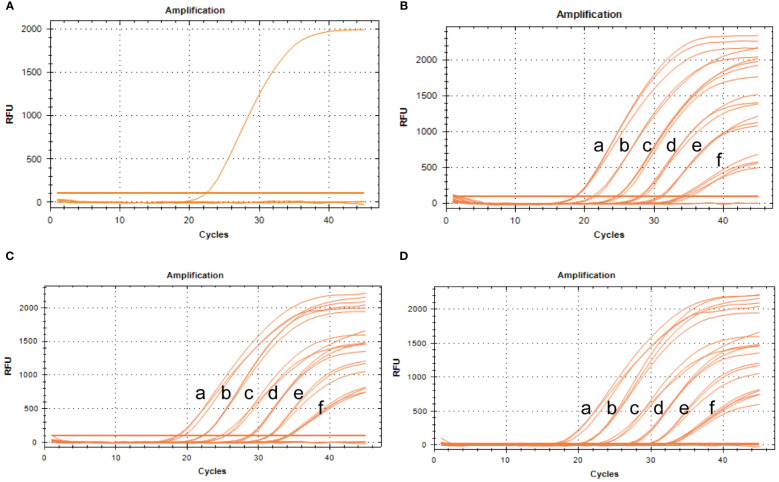
Specificity and repeatability analysis of the BRBV-sheep qRT-PCR assays. **(A)** Specificity assay amplification results. Only BRBV-sheep nucleic acid demonstrated a positive fluorescence signal, while no positive signal was observed with nucleic acid from other pathogens. **(B)** Intra-assay reproducibility of qRT-PCR assay (First repetition). a to f represents the templates, with concentrations ranging from 1 × 10^6^ to 1 × 10^1^ copies/μL. **(C)** Intra-assay reproducibility of qRT-PCR assay (Second repetition). a to f denotes the templates, with concentrations ranging from 1 × 10^6^ to 1 × 10^1^ copies/μL. **(D)** Intra-assay reproducibility of qRT-PCR assay (Third repetition). a to f depicts the templates, with concentrations ranging from 1 × 10^6^ to 1 × 10^1^ copies/μL.

To assess specificity, the BRBV-sheep and other pathogens—IBRV, BPIV-3, Pasteurella multocida serotype A, Mannheimia haemolytica, Pasteurella multocida serotype B, Mycoplasma bovis, and Klebsiella pneumoniae—were tested. No cross-reactivity was observed between IBRV, BPIV-3, Pasteurella multocida serotype A, Mannheimia haemolytica, Pasteurella multocida serotype B, Mycoplasma bovis, or Klebsiella pneumoniae. Furthermore, non-specific amplification wasn’t observed in the negative samples (**Figure 8A**). These findings confirm the specific of qRT-PCR.

### 3.8 Reproducibility of TaqMan-based qRT-PCR for BRBV sheep

To evaluate intra-assay and inter-assay reproducibility, six 10-fold diluted positive control plasmids were selected, and the experiment was conducted in triplicate. **Figures 8B–D** presents the results. The coefficients of variation within the intra-assay were <2%. The amplification curves of the same concentration gradient were tightly clustered, with no obvious gap in the cycle number. **Table 3** shows the inter-assay results, showing coefficients of variation <2%. These findings suggest that qRT-PCR was stable.

**Table 3:** The repeatability of the developed qRT-PCR of BRBV-sheep.

Standard Copies/μL	Intra-assay repeatability of CT-value			Inter-assay repeatability of CT-value		
	Mean	SD	CV (%)	Mean	SD	CV (%)
1 × 10^6^	18.60	0.15	0.26	18.56	0.18	0.16
1 × 10^5^	21.94	0.18	0.24	21.67	0.17	0.88
1 × 10^4^	24.00	0.11	0.25	24.20	0.19	0.74
1 × 10^3^	28.32	0.16	0.42	28.19	0.13	0.56
1 × 10^2^	30.35	0.16	0.59	30.49	0.14	1.11
1 × 10^1^	33.99	0.18	0.50	33.95	0.19	0.23

The intra-assay and inter-assay repeatability was assessed using 10-fold serial dilutions of DNA standard ranging from 1 × 10^6^ to 1 × 10^1^ copies/μL.

### 3.9 Clinical samples

Between February 2019 and March 2022, 592 clinical samples (nasal swabs) were collected in Inner Mongolia, China, for analysis using the developed qRT-PCR to validate its utility. Based on the assay outcomes, 327 samples tested positive via TaqMan-based qRT-PCR, while 287 samples yielded positive results using RT-PCR (**Table 4**). Consequently, the positivity rates detected via the conventional RT-PCR and TaqMan-based qRT-PCR were 33.78% (200/592) and 42.72% (226/592), respectively. This finding shows that the TaqMan-based qRT-PCR assay developed in this study exhibited higher sensitivity and accuracy than the conventional one.

**Table 4:** Clinical sample information.

Year	Location	No. of samples	No. of RT-PCR		No. of TaqMan-qPCR	
			P	N	P	N
2019	Ji’ning (slaughterhouses)	46	19	27	21	25
	Hohhot (slaughterhouses)	65	29	36	30	35
	Horinger, Hohhot (house feeding)	38	15	23	16	22
	Tumd Zouqi, Hohhot (house feeding)	30	10	20	14	16
2020	Ji’ning (house feeding)	48	16	32	18	30
	Hohhot (slaughterhouses)	57	25	32	28	29
	Ulan Hot (grazing feeding)	59	10	49	11	48
	Bayan Nur (house feeding)	62	28	34	30	32
2021	Otog Qi, Ordos (grazing feeding)	53	12	41	15	38
	Jungar Qi, Ordos (house feeding)	40	17	23	19	21
	Ongniud Qi, Chifeng city (grazing feeding)	62	12	50	16	46
	Xilinhot (grazing feeding)	32	7	25	8	24

Moreover, the sheep housed and fed under conditions—with their health status known—exhibited a higher rate of positive detection (44.50%; 97/218) than those maintained under traditional grazing conditions (24.27%; 50/206). Further, these traditionally grazed sheep shared the same pasture as the cattle; however, their rate of BRBV positivity detection was relatively low. A greater percentage of positive detections was observed among sheep transported over long distances in slaughterhouses (47.02%; 79/168) than among house-fed sheep (44.50%; 97/218).

## 4 Discussion

In recent years, the emergence of next-generation sequencing (NGS)-based viral metagenomics has offered a potent methodology to comprehensively and impartially identify known and unknown viruses in animals on a large scale (Hause et al., 2015; Ng et al., 2015; Mitra et al., 2016; Zhang et al., 2019; Zhang et al., 2020; Zhai et al., 2021; Zhang et al., 2021; Ambrose et al., 2023; Brito et al., 2023). In this present study, viruses belonging to various families in nasal swabs collected from sheep were successfully detected. Certain viruses, including BRBV, Picobirnavirus TFFN-2015, and Tusavirus 1, had not been previously detected in Chinese sheep. This study highlighted that sheep can be highly susceptible to BRBV infection, a prevalence not previously reported.

In this study, we conducted full-length sequencing of the BRBV sheep genome. After annotating the characteristics and conducting a comparative analysis of the sequence alignment between BRBV sheep and other aphthoviruses and BRVs, several distinctive attributes were identified within its genetic composition. The BRBV-sheep sequence exhibited conserved features characteristic of the BRBV species. Among FMDV capsid proteins, VP1 is the sole protein exhibiting an arginine-glycine-aspertate (RGD) tripeptide motif in its G–H loop, responsible for binding cellular integrins (McKenna et al., 1995; Jackson et al., 1997; Rieder et al., 2005; Rai et al., 2015). Structural modeling and sequence alignment analysis revealed the absence of an essential cellular receptor-binding site in the remaining three aphthoviruses (BRAV, BRBV, and ERAV). This finding suggests a distinct mechanism for cell attachment employed by these viruses. The accuracy of phylogenetic tree inference was validated by constructing neighbor-joining phylogenetic trees utilizing the highly conserved 3Dpol region. The clustering of BRBV-infected sheep with BRBV isolates further supports its classification within this species. Additionally, the unique cluster formed by BRBV sheep suggests a notable evolutionary divergence among these isolates. Assessing selection pressure is valuable for identifying shared lineages among rapidly evolving organisms (Kumar et al., 2004; Beaudeau et al., 2010; Chi et al., 2013; Yuvaraj et al., 2013). The utilization of various analytical techniques in this study bolstered the confidence in concluding that no positive selection exists between BRBV sheep and other aphthoviruses. It is not surprising that BRBVs have a significant number of positive selection breakpoints; this further supports their shared origins. As anticipated, the limited evidence of distinct positive selection sites aligns with the notion of a separate but parallel lineage among the aphthoviruses examined in this study. Various algorithms within specific software packages were used to detect potential recombination breakpoints. Our recombination analysis among Aphthoviruses and BRVs suggests that FMDV, BRBV, and BRAV may undergo inter-species recombination. However, the confirmation of multiple recombination breakpoints in both BRBV species substantiated the role of homologous recombination in shaping the diversity of Picornaviridae.

Developing an accurate RT-qPCR assay is crucial for precisely detecting and quantifying BRBV-sheep. Ultimately, we introduce a widely utilized TaqMan-based quantitative real-time PCR method for diagnosing BRBV-sheep infection in sheep. Through comprehensive analytical specificity testing, we confirmed that the primers and probes utilized in this TaqMan-based quantitative real-time PCR demonstrated specific reactivity towards the BRBV sheep pathogen. Consequently, this assay demonstrated a high reliability in detecting coinfections with various pathogens.

**Figure 9:**
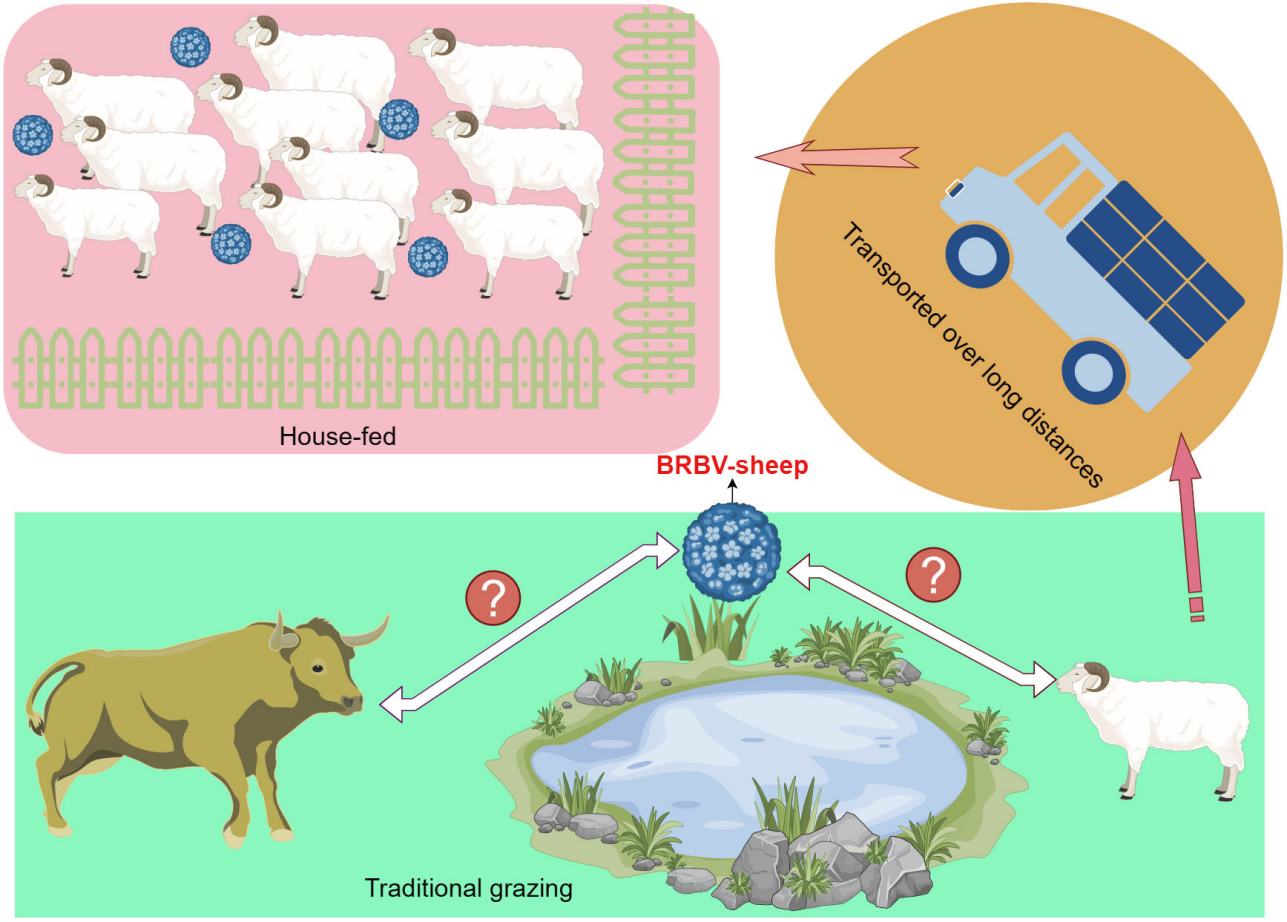
Schematic representation of BRBV-sheep virus spread, encompassing transmission in traditional grazing environments and via airborne transmission among sheep within home pens and among home pens in a sheep feedlot.

Our epidemiological survey on sheep in Inner Mongolia, China, revealed a 42.72% prevalence of BRBV infection. Considering these findings, we hypothesized the potential transmission of BRBV from cattle to sheep during traditional grazing due to shared pasture. Numerous studies have established a high prevalence of BRBV infection among cattle in China (Rai and Rieder, 2012; Uddowla et al., 2013; Hause et al., 2015; Mitra et al., 2016; Blomstrom et al., 2017; Xie et al., 2021; Zhai et al., 2021; Zhang et al., 2021; Bhattarai et al., 2022; Zhou et al., 2023). However, our findings were inconsistent with our hypothesis, as the prevalence of BRBV-sheep among traditionally grazed sheep stood at only 24.27%. In contrast, housed sheep exhibited a higher prevalence of 44.50%. This may be attributed to the farm owners who established conditions conducive to widespread transmission by introducing BRBV-positive sheep onto their farms (**Figure 9**). Most house-fed sheep in Inner Mongolia are typically purchased from herders who traditionally graze them. Subsequently, these sheep are fed commercial full-price diets for 3 months before being slaughtered. However, the central question regarding the relationship between BRBV and sheep remains unanswered. We were uncertain whether the virus was transmitted from cattle to sheep or vice versa. Therefore, addressing this question may necessitate collecting substantial genetic data from diverse BRBV-sheep strains.

## 5 Conclusions

The molecular data presented in this study provide evidence that BRBV-sheep infections are ubiquitous in the upper respiratory tract of sheep. While our findings do not establish the role of BRBV in the pathogenesis of upper respiratory tract infections in sheep, in more than half of the clinical samples from BRBV-sheep-positive sheep, no other viruses were detected using metagenomic sequencing. Therefore, further investigation is warranted to determine whether BRBV-sheep are non-pathogenic commensal organisms. In conclusion, this study enhances our knowledge of BRBV infection in sheep. It contributes to the understanding of the epidemiology of these viruses.

## Data availability statement

All raw sequences files in this study were submitted to the NCBI Sequence Read Archive (SRA) and are associated with BioProject accession PRJNA1040299 (https://www.ncbi.nlm.nih.gov/bioproject/PRJNA1040299/).

## Ethics statement

The animal study was approved by the Institutional Animal Care and Use Committee at Inner Mongolia Agricultural University, China. The study was conducted in accordance with the local legislation and institutional requirements.

## Author contributions

YZ: Writing – review & editing, Writing – original draft, Visualization, Validation, Supervision, Software, Resources, Project administration, Methodology, Investigation, Funding acquisition, Formal analysis, Data curation, Conceptualization. YL: Writing – review & editing, Visualization, Validation, Supervision, Software, Resources, Methodology, Investigation, Formal analysis, Data curation, Conceptualization. LQ: Writing – review & editing, Visualization, Software, Resources, Methodology, Formal analysis. TH: Writing – review & editing, Validation, Investigation, Data curation, Resources. PW: Writing – review & editing, Software, Methodology, Investigation, Data curation. YaW: Writing – review & editing, Validation, Resources, Methodology, Data curation. CW: Methodology, Data curation, Writing – review & editing, Formal analysis, Conceptualization. YoW: Methodology, Formal analysis, Data curation, Writing – review & editing, Validation, Software, Investigation. XW: Software, Methodology, Investigation, Data curation, Writing – review & editing. LH: Investigation, Writing – review & editing, Supervision, Formal analysis. YB: Formal Analysis, Writing – review & editing, Methodology. ZZ: Methodology, Formal analysis, Writing – review & editing, Visualization, Validation, Supervision, Software, Resources, Investigation, Data curation. WZ: Visualization, Validation, Supervision, Software, Resources, Methodology, Investigation, Formal analysis, Data curation, Writing – review & editing, Writing – original draft, Project administration, Funding acquisition, Conceptualization.

## Supplementary material

Supplementary Figure 1 Alignment of aphthovirus polyproteins: The alignment and feature annotation were conducted using the BRBV-sheep strain as a template, marked as OBRV/1–2283. The sequences of BRBV (GenBank: UFQ04588.1, AJE25834.1, and QBP41068.1), BRAV (GenBank: YP 009352243.1), and FMDV (GenBank: AAT01740.1) were aligned with BRBV-sheep (GenBank: OQ547742.1). The start of each protein sequence (N-terminus of) is marked, followed by the protein identification. Yellow highlights indicate functionally critical residues (active site residues). (-) denotes a gap introduced in the alignment. Cyan highlights denote functionally important protein residues other than active site residues. Red highlights denote the RGD cell surface receptors.
