# Supplementary material for: Discovery and characterization of BRBV-sheep virus in nasal swabs from domestic sheep in China
Source: Front Cell Infect Microbiol. 2024 Jun 28;14:1380708. doi: 10.3389/fcimb.2024.1380708 (PMC11239340; doi:10.3389/fcimb.2024.1380708)
Supplement: Supplementary Figure 1 — Alignment of aphthovirus polyproteins: The alignment and feature annotation were conducted using the BRBV-sheep strain as a template, marked as OBRV/1–2283. The sequences of BRBV (GenBank: UFQ04588.1, AJE25834.1, and QBP41068.1), BRAV (GenBank: YP 009352243.1), and FMDV (GenBank: AAT01740.1) were aligned with BRBV-sheep (GenBank: OQ547742.1). The start of each protein sequence (N-terminus of) is marked, followed by the protein identification. Yellow highlights indicate functionally critical residues (active site residues). (-) denotes a gap introduced in the alignment. Cyan highlights denote functionally important protein residues other than active site residues. Red highlights denote the RGD cell surface receptors. [file DataSheet_1.pdf]

Fig. S1

Lpro

```
OBRV/1-2283                     --------MTTERLLHHLLIDTIHKKINKINKMALELGFNNTLVT-ADLDGNKKIIHGLP
UFQ04588.1/1-2283BRBV           --------MTTERLLHQLLVHTIHNKINKINNMALELGFNNTLTT-ADLDGNEKIIHGLP
AJE25834.1/1-2291BRBV           MFFPRAHFMTTERLLHQLLIHTIHKKINKRNNMALELGFNNTLKT-TDLDGNEKIIHGLP
QPB41068.1/1-2274BRBV           --------MTTERLLHHLLIDTIHKKINKTNNMALELGFNNTLVT-ADLDGNEKIMHGLP
YP_009352243.1/1-2218BRAV       ---------------------------------------MKTTIMEFTKYNGGQKVSIHGLL
AAT01740.1/1-2311FMDV           -------MNTTDCFIA--LLHAI-RKI----KARLLLRTQEKMEF-TLYNGEKKTFYSRP

OBRV/1-2283                     NKHDNCWLNALMQMTNWVGEGFFRNTYDNP-DLIPQTMKFLSEYTGI-DLSYGGPPSIVL
UFQ04588.1/1-2283BRBV           NRHDNCWLNSLMQMTNWVGEGFFKNTYDNP-DLIPQTIKFLTEYTGI-DLSYGGPPSIVL
AJE25834.1/1-2291BRBV           NRHDNCWLNALMQMTNWVGEGFFKNTYDNP-DLIPQTIKFLTEYTGI-DLSYGGPPSIVL
QPB41068.1/1-2274BRBV           NRIDNCWLNSLMQMTNWVGEGFFRETYDNP-DLIPQTIKFLTDYTGI-DLSYGGPPSIVL
YP_009352243.1/1-2218BRAV       NDNDNCWLNSLAQLANYMDSVFFDSYYNGN-SSSMDEILQLQTLTGIQDLTYGGPPSIVL
AAT01740.1/1-2311FMDV           NNHDNCWLNTILQLFRYVDEPFFDWVYESPENLTLEAIRQLEEVTGL-ELHEGGPPALVI

OBRV/1-2283                     YKIRDLLDTKVGTSKEPGDYVVSCQGVYCLADMQAGVFMEGEEHAVFYACTALGWIKVDD
UFQ04588.1/1-2283BRBV           YKIRDLLDTKVGTSKEPGDYVVSCQGNYCLADMQAGVFMEGEEHAVFYACTALGWIKVDD
AJE25834.1/1-2291BRBV           YKIKDLLDTKVGTSKEPGDYVVSCQGVYCLADMQAGVFMDGEEHAVFYACTALGWIRVDD
QPB41068.1/1-2274BRBV           YKIRDLLDTKVGTSKEPGDYVVSCQGTYCLADMQAGVFMEGEEHAVFYACTALGWVKIDD
YP_009352243.1/1-2218BRAV       YKIKDYLDFTVGTPSNPGQVCVTCGCDMTLADMHAGIFLDGDEHAVFYFRSEDGWVCVDD
AAT01740.1/1-2311FMDV           WNIKHLLHTGIGTASRPSEVCMVDGTDMCLADFHAGIFLKGQEHAVFACVTSNGWYAIDD
```

VP4

```
OBRV/1-2283                     ETITRCIPDPADVLVFVPWDREAI-CDYDTEFFNQVYVFGAGSSKPQSGNINESGNSGSI
UFQ04588.1/1-2283BRBV           ENIVRCIPDPANVLVFVPWDREAI-CDYDTEFFNQVYVFGAGSSKPQSGNVNESGNSGSI
AJE25834.1/1-2291BRBV           ENITRCIPDPANVLVFVPWDRETI-CDYDTEFFNQVYLFGAGSSKPQSGNVNESGNSGSI
QPB41068.1/1-2274BRBV           ENITRCIPDPANVLVFVPWDRETI-CDYDAEFFNQVYVFGAGSSKPQSGNVNESGNSGSI
YP_009352243.1/1-2218BRAV       DRWYFATPDPAHVLVFVPFDDEPM-GQDSEIVFATCYAFGGGQSKPQSGNMNQSGNSGSV
AAT01740.1/1-2311FMDV           EDFYPWTPDPSDVLVFVPYDQEPLNGEWKTKVHKR--LFGAGQSSPATGSQNQSGNTGSI

OBRV/1-2283                     VNNYYMQQYQNSIDATIGDKTTEGGSGSGDTAGSATHNNTTKQDKDKD--DWFSSLMSGV
UFQ04588.1/1-2283BRBV           VNNYYMQQYQNSIDATVGDKTTEGGSGSGDTAGSATHNNTTKEDKGKD--DWFSSLMSGV
AJE25834.1/1-2291BRBV           VNNYYMQQYQNSIDATIGDKTTEGGSGSGDTAGSATHNNTTKHDKEKD--DWFSSLMSGV
QPB41068.1/1-2274BRBV           VNNYYMQQYQNSIDATVGDKTTEGGSGSGDTAGSATHNNTTKQDKDKD--DWFSSLLSGV
YP_009352243.1/1-2218BRAV       VNNYYMQQYQNSIDTTLGDKPVIGGSGQGDTAGSATHNQNTTSPSGGGGMDWFGHLTNLA
AAT01740.1/1-2311FMDV           INNYYMQQYQNSMDTQLGDNAISGGSNEGSTDTTSTHTNNTQNN------DWFSRLASSA
```

VP2

```
OBRV/1-2283                     GSAIPGAVVGLLADKKTEETTKLEDRIMSTRHRTDITTTQSSVGVISGYAFEE-KDQVVA
UFQ04588.1/1-2283BRBV           GSAIPGAVVGLLADKKTEETTKLEDRIMSTRHRTDITTTQSSVGIVSGYALEE-KDQIVA
AJE25834.1/1-2291BRBV           GSALPGAVVGLLADKKTEETTKLEDRIMSTRHRTDITTTQSSVGVVNGYALGE-ADQIVA
QPB41068.1/1-2274BRBV           GSALPGAVVGLLADKKTEETTNLEDRIVSTRHGTDITTTQSSVGVTYGYAIRE-KDDIVA
YP_009352243.1/1-2218BRAV       SNVLPAAI-GLLADHKTEETTKLEDRILVTRVGPTTQTTQSSVGVVQGYGGPEPQDTTTL
AAT01740.1/1-2311FMDV           FSGLFGA---LLADKKTEETTLLEDRILTTRNGHTTSTTQSSVGVTYGYAVTEDAVSGPN

OBRV/1-2283                     ASGTHEHVTHVSRIYMKKLFKWSISDQVATYHAYALPDQILSEAKDYKNLLSAYALYRNG
UFQ04588.1/1-2283BRBV           ASGTHEHVSHVSRIYMKKLFKWSTAEQVATYYSYTLPDQILPEAKDYKNLLSSYALYRNG
AJE25834.1/1-2291BRBV           ASGTHEHVSHVSRIYMKKLFTWSTNISAGTYYSYPLPETILSEAKDYRNLLKAYALYRNG
QPB41068.1/1-2274BRBV           ASGTHTKAGHVTRIYSKTLGQWSLTDNVGHRWTMNLPSDILAEARDYADLLKNYALYRNG
YP_009352243.1/1-2218BRAV       AAGLAVEVPSAQRFYDIGTWDWSTTSAAGHTKRYPLPASLRKGA--FANLAKTYVLMQNG
AAT01740.1/1-2311FMDV           TSGLETRVIQAERFFKKHLFDWTQDLSFGHCHYLELPSEHKGV---YGGLMDSYAYMRNG

OBRV/1-2283                     WEVHVSVVSTIYHSGCLVVALVPEFTSGQQTGKTYDPNFAQLTLFPHQFINLRTNTTASI
UFQ04588.1/1-2283BRBV           WEVHVSVVSTIYHSGCLVVAMVPEFTPGTTSGRTYDPNFAQLTLYPHQFINLRTNTTASI
AJE25834.1/1-2291BRBV           WEVHVSVVSTMYHSGCLVVAMVPEFSETTQNGQTFDPHFAQLTLYPHQFINLRTNTTASI
QPB41068.1/1-2274BRBV           WEVHVSVTATIYNSGCLVVAMVPEY---NFSPNGFNAEFAQITLYPHQLLNLRTNATASI
YP_009352243.1/1-2218BRAV       WEIVVSAQGTLSHGGMLMVAMVPELNFKTES-DAQPADYRQYTVFPHQMINPRTNTTAHI
AAT01740.1/1-2311FMDV           WDIEVTAVGNQFNGGCLLVALVPELKS-LDTRQKY-----QLTLFPHQFINPRTNMTAHI
```

VP3

```
OBRV/1-2283                     RVPYVGATDMDDHRLHSTWTLIVGIVVPLKIHNGNSDLTAIDVRASITPTNVKVAGPLPN
UFQ04588.1/1-2283BRBV           RVPYVGATDMDDYRLHSAWTLIVGIVVPLKIHNGNSDLTAIDVRASITPTNVKVAGPLPN
```

Fig. S2

```
                                                                         VP3
AJE25834.1/1-2291BRBV          RVPYVGATDMDDHRLHQAWTLLVGIVVPMQIHNGNSDLTAIEVRASITPTNVKVAGPMPN
QPB41068.1/1-2274BRBV          RVPYVGATDMDDHRLHKPWTLVVGVVAPLQ--DGGSDLTVVDIRVSVTPLSVHVAGPMPN
YP_009352243.1/1-2218BRAV      RVPYVGAFSMEDVNKHNAYTLVLVVVSPLT--AGGHTKTVVKVKVTAAPIDVRVVGETPE
AAT01740.1/1-2311FMDV          NVPFVGVNRYDQYALHKPWTLVVMVVAPLTVKTGGSE--QIKVYMNAAPTYVHVAGELPS

OBRV/1-2283                    KQGIIPVAVKSGYSGFSTTSPITADPIYGQVVNPPRRHIPGRFTNFLDVADKCPTMARFV
UFQ04588.1/1-2283BRBV          KQGIIPVAVKSGYSGFSTTSPITADPIYGQVVNPPRKYIPGRFTNFLDVADKCPTMARFV
AJE25834.1/1-2291BRBV          KEGIVPVAVKSGYSGFSTTSPITADPVYGQVVNPPRRYIPGRFTNFLDVCDACPTMARFT
QPB41068.1/1-2274BRBV          KQGILPVATKAGYSGFTTTGPLTADPVYGNVVNPPRRHIPGRFTNFLHVADACPTMARFQ
YP_009352243.1/1-2218BRAV      RQGLVPLAPHVGYGGFKTTAPITADPVCGGVYNPPRQDMPGRFTNFLQVAQVCPTFGKVG
AAT01740.1/1-2311FMDV          KEGIVPVACVDGYGNMVTTDPKTADPVYGKVFNPPRTNLPGRFTNFLDVAEACPTFLRFG

OBRV/1-2283                    S--KPSVTTQQGASEVLLSTIDVSLVSHELSFTYLAGLASLYAQYRGSINMHCIYTGFAS
UFQ04588.1/1-2283BRBV          S--KPSVTTQQGASEVLLSTIDVSLVSHELSYTYLAGLASLYAQYRGSINMHCIYTGFAS
AJE25834.1/1-2291BRBV          N--KPSVSTRSSASEVLLATIDVSLVSHELSYTYLAGLASLYAQYRGSINMHCIYTGFVS
QPB41068.1/1-2274BRBV          N--KPTLTTSSDPHK-LLATIDVSLMSHEMSYTYLAGLSALYAQYRGTINVHFVYTGPVS
YP_009352243.1/1-2218BRAV      SGATPYFVTQTD-NDVLLSTIDVSLTSYEMSSTFLAGLAQFYAQYRGTINVHFVFTGSVN
AAT01740.1/1-2311FMDV          E--VPFVKTVNSGDR-LLAKFDMSLAAGHMSNTYLAGLAQYYTQYSGTMNIHFMFTGPTD

OBRV/1-2283                    DKAKFLLAYIPPGAEQPRTLSEAQHCITLEWDTGLNSETVFNIPYLSQTYYTSTHSSDAD
UFQ04588.1/1-2283BRBV          DKAKFLLAYVPPGAEQPRTLSEAQHCITLEWDTGLNSETVFNIPYLSQTYYTSTHSSEAD
AJE25834.1/1-2291BRBV          DKAKFLLVYVPPGADKPTTLSEAQHCITYEWDTGLNSEAVFNIPYISQTYYTSTHSSEAD
QPB41068.1/1-2274BRBV          DKAKFLVVYIPPGATVPDNLSQAQHCVTLEWDSGLNSEAVFSVPYVSQTYYTSTYSGEAD
YP_009352243.1/1-2218BRAV      DKARFRVVFVPPGTDPPTTAVDASLHIHSDWDSGLNSEFVYPVPYVSPTPYTTTHGAEAD
AAT01740.1/1-2311FMDV          AKARYMVAYIPPGMTPPTDPERAAHCIHSEWDTGLNSKFTFSIPYLSAADYAYTASDVAE

                                                                                VP1
OBRV/1-2283                    IGNVSGRVQIFQVTE-TSTPAELLVLFSSGSDFQLRCPVEPV--KQITDVGETGKYTTLD
UFQ04588.1/1-2283BRBV          IGNVAGRVQIFQVTE-TSTPAELLVLFSSGSDFQLRCPIEPV--KQVTDVGETGKYITLD
AJE25834.1/1-2291BRBV          IGNVAGRVQIYQVTH-VSTTSELLILFSSGSDFQLRCPVEPV--KQVTDVGESGKYVTLD
QPB41068.1/1-2274BRBV          IGNVSGWLQIYQVTR-AKTGNQLLVMFSSGTDFQLRCPIEPV--RQVTDVGESGHYKTLD
YP_009352243.1/1-2218BRAV      QATVNGWIQIYQLDA-TASNLAVTVAFSAGPDFELRFPCEPVHYEVTTDVGESGVGHDAT
AAT01740.1/1-2311FMDV          TTSVQGWVCIYQITHGKAEGDALVVSVSAGKDFEFRLPVDAR--RQTTTAGESADPVTTT

OBRV/1-2283                    ATQQNGFKANTFRLHTDVAFALDRYTQLATIKGNRVNTHNTATNLDPTKLPLNTFVKKLV
UFQ04588.1/1-2283BRBV          ATQQNGFKANTFRLHTDVAFALDRYTQLATIKGNSADAHNTTTNLDPTKLPLNTFVKKLV
AJE25834.1/1-2291BRBV          ATQQHGDRRPAFRLHTDVSFALDRYTQLMNTGGNRTNTDKKSINLNPTQLPPDTFIKKLV
QPB41068.1/1-2274BRBV          ARQQQGDKQPTFRLHTDVAFALDRFTQLKTTSGNKEDTHNNFTNLDLMTLPNGTFVKTLL
YP_009352243.1/1-2218BRAV      VERLGGVDAPSFRTHTDVSWVLDRYAIAAKVAGYKASLGAGFV-LDPVALPTTSMMGQLL
AAT01740.1/1-2311FMDV          VENYGGETQAARRLHTDVAFVLDRFVKLTQPKNTQT--------LDLMQIPSHTLVGALL

OBRV/1-2283                    DSCTYYFSDLEVTVNAKGTVPAWANVTWHPVSAPRTFTNNNYPDEDNDFMTVNSNASVGF
UFQ04588.1/1-2283BRBV          DSCTYYFSDLEVTVNAKGTVPAWANVTWYPVSAPRTFTNNNYPDEENDFMTVNSNASVGF
AJE25834.1/1-2291BRBV          DSCTYYFSDLEVTVNAKGDVPPWANVTWHPVASPRTFTNDNFPDEENDFMTVNSNVSVGF
QPB41068.1/1-2274BRBV          SSCTYFFSDLEISVNARGQVPPWAHVTWYPVSAPRSFTQNLYDDDDNDFMTLSSNVSVGF
YP_009352243.1/1-2218BRAV      RAATYYFADLELAVVPRGQPGEYAMVKWLPVGTPFDLADTGL--DGLALQGLDSTCSVGF
AAT01740.1/1-2311FMDV          RSATYYFSDLEVALVHTGS------VTWVPNGAPKDALDN------------HTNPTAYQ

OBRV/1-2283                    TGPSGGGSIATFAIPYTSIYRVLPTKYSGRTQFTVVDNFKA-LSYTGFGEI--------T
UFQ04588.1/1-2283BRBV          TGPSGSGSIATFAIPYTSIYRVLPTKYSGRTQFTLVDNFKA-LSYTGFGEI--------T
AJE25834.1/1-2291BRBV          TGPAGGGTVATFAIPYTSLYRVLPTRYSGRTQFTQQDGFKA-LSYVGFGEI--------T
QPB41068.1/1-2274BRBV          QGPGGGSSVATFAIPYTSLYRVLPTTYNGTTFHGGNARTKQ-FNHTGFGEI--------F
YP_009352243.1/1-2218BRAV      TGSAGNGSAAVMAIPYNSPMRVIPTVYAGTTQYTHTSPARP--GTANYGLI--------F
AAT01740.1/1-2311FMDV          KQP-----ITRLALPYTAPHRVLATVYNGKTTYGEQSTRHGDLATLAQGVSNRLPTSFNY

                                                                                      2A
OBRV/1-2283                    VAGLENVFHRVLIRMKRTEMYCPRPL-YPTTSVSTVSTRRKFPLVSESVPPGKQGATNFD
UFQ04588.1/1-2283BRBV          VAGLENTNHRILIRMKRTEMYCPRPL-YPSTTVNTVSTRRKFPLVSENTPLKKQGATNYS
AJE25834.1/1-2291BRBV          VAGLEQTSHRVLIRMKRTEMYCPRAL-YPSNTLTKITERRKLVLVSESVNPSKQGAKNFD
QPB41068.1/1-2274BRBV          VTGLDDVKHRVLVRIKRAELYCPRFI-QAPHNSSALSSRYKTTLAGAIV---KQGATNFS
YP_009352243.1/1-2218BRAV      VIGDSGVTFRVMYRLKRTELYCPRPLVYRQKNTVTFGKRQKFKLAGIDK---ESGISNKD
AAT01740.1/1-2311FMDV          GAVKADTITELLIRMKRAETYCPRPL----LALDTTQDRRKQEIIAP-----EKQALNFD
```

Fig. S3

```
                                                        2B
OBRV/1-2283                    LLKRAGDIESNPGP-------TAFSKLID---------------DFGCLSSSMEEIARHI
UFQ04588.1/1-2283BRBV          LLQQAGDVETNPGP-------SAFSKLIE---------------DFGCLSNSMEEIARHI
AJE25834.1/1-2291BRBV          LLKLAGDVESNPGP-------TAFSKLID---------------DFGCLSNSMEEIARHI
QPB41068.1/1-2274BRBV          LLKQAGDVESNPGP-------TAFSKLID---------------DFGCLSNSMEEIARHI
YP_009352243.1/1-2218BRAV      LLLQAGDVETNPGP-------GVFSQFAD---------------LAASATQDFHNLTEGI
AAT01740.1/1-2311FMDV          LLKLAGDVESNPGPFFFSDVRSNFSKLVDTINQMQEDMSTKHGPDFNRLVSAFEELATGV

OBRV/1-2283                    GDFKVMMKGAGPWYKAFKYLWKVATVIVAITRTKDAVLVGMLLADIGLEVFDTRVMMDSL
UFQ04588.1/1-2283BRBV          GDFKAMMKGAGPWYKAFKYLWKVATIIVTITRTKDTVLVGMLLADIGLEIFDTRVMMDSL
AJE25834.1/1-2291BRBV          GDFKAMMKGAGPWYKAFKYLWKVATVIVTITRTKDAVLVGMLLADIGLEVFDTRVMMDSL
QPB41068.1/1-2274BRBV          GDFKTMMRGAGPWYKAFKYLWKVATVIVAITRTRDTVLVGMLLADIGLEVFDTRIMMDSL
YP_009352243.1/1-2218BRAV      LELKNTLKGAGPWYKAFKYIWKLATLVVTAFRTQDPVVIAMQLADLGIEIFEAEVLVRGL
AAT01740.1/1-2311FMDV          KAIRTGLDEAKPWYKLIKLLSRLSCMAAVAARSKDPVLVAIMLADTGLEILDSTFVVKKI

                                                                                    2C
OBRV/1-2283                    VDRFKPYFHVNPPKFDFK-VEVL----DKVRDFFATEEDEEEFDDTN-PFKQISLKNIND
UFQ04588.1/1-2283BRBV          VDRFKPYFHVDPPKFDFK-TEVL----DKVRDFFATGEEEEEFDDTN-PFKQISLKSVND
AJE25834.1/1-2291BRBV          VDRFKPYFHVNPPKFDFK-TEVL----DKVRDFFANDDEEEEFDDTN-PFKQISLKNVND
QPB41068.1/1-2274BRBV          VDRFKPYFHVDPPKFDFK-TEIL----DKVRDFFAGGEEEEEFDDTN-PFKQISLKNLND
YP_009352243.1/1-2218BRAV      AQKMSEQFQTPPPKFEFKYSELI----EKAEQIF------EDFDDDEAPEKQFSMKRLND
AAT01740.1/1-2311FMDV          SDSLSSFFHVPAPVFSFGAPTLLAGLVKVASNFFRS--TPEDLERAE---KQLKARDIND

OBRV/1-2283                    VFNLVKNGQWLMGFFLSLRDWFRTWLDSEEKFITYHDLVPQIIEHQERLLVPDEYAEAHN
UFQ04588.1/1-2283BRBV          IFNLVKNGQWLMSFFLSLRDWFRTWLESEEKFITYHDLVPQIIEHQERLLVPDEYAEAHN
AJE25834.1/1-2291BRBV          IFNLVKNGQWLMSFFLSLRDWFRTWLESEEKFITYHDLVPQIIEHQERLLVPDVYAEAHN
QPB41068.1/1-2274BRBV          IFNLVKNGQWLMSFFLSLRDWFRAWLDSEEKFITYHDLVPQIIEHQERLLIPDEYAEAHN
YP_009352243.1/1-2218BRAV      IFSFLKNGEWLIKFFLSIRSWVRTWLKQEETVMSYNDLVPKIIQKQLELKEPSTFAQAKN
AAT01740.1/1-2311FMDV          IFAILKNGEWLVKLILAIRDWIKAWIASEEKFVTMTDLVPGILEKQRDLNDPSKYKEAKE

OBRV/1-2283                    WLERKREVLLQANQYALAKLCEPKVGPPPETRPEPVVILFRGDSGQGKSFLSNLIAQALS
UFQ04588.1/1-2283BRBV          WLERKREVLLQANQYALAKLCEPKVGPPPETRPEPVVILFRGDSGQGKSFLSNLIAQALS
AJE25834.1/1-2291BRBV          WLERKREVLLQANQYALAKLCEPKVGPPPETRPEPVVVLFRGDSGQGKSFLSNLIAQALS
QPB41068.1/1-2274BRBV          WLERKREVLLQANQYALAKLCEPKVGPPAETRPEPVVVLFRGDSGQGKSFLSNLIAQALS
YP_009352243.1/1-2218BRAV      WLVRQREILLTAGQKDLAQLCEVKVKEPVTGRPEPVVLVLRGKSGQGKSFMANILASAIS
AAT01740.1/1-2311FMDV          WLDNARQACLKSGNVHIANLCKVATPAPSESRPEPVVVCLRGKSGQGKSFLANVLAQAIS

OBRV/1-2283                    KLLTGRVDSIWSCPPDPDHFDGYRGQKVVIMDDLGQNPDGTDFKYFAQMVSTTAFIPPMA
UFQ04588.1/1-2283BRBV          KLLTGRVDSIWSCPPDPDHFDGYRGQKVVIMDDLGQNPDGTDFKYFAQMVSTTAFIPPMA
AJE25834.1/1-2291BRBV          KLLTGRVDSIWSCPPDPDHFDGYRGQKVVIMDDLGQNPDGTDFKYFAQMVSTTAFIPPMA
QPB41068.1/1-2274BRBV          KLLTGRPDSIWSCPPDPDHFDGYRGQKVVIMDDLGQNPDGTDFKYFAQMVSTTAFIPPMA
YP_009352243.1/1-2218BRAV      RMLTGKPDSVWSCPPDPTYFDGYRGQSVVIMDDLGQNPDGKDFKYFAQMVSSTAFVVPMA
AAT01740.1/1-2311FMDV          THFTGKTDSVWYCPPDPDHFDGYNQQTVVVMDDLGQNPDGKDFKYFAQMVSTTGFIPPMA

OBRV/1-2283                    ALEDKGKVFNSPVIIATTNMHEHFTPKTMACPGALLRRFTFDYVLAARKPYIREKTETLN
UFQ04588.1/1-2283BRBV          ALEDKGKVFNSPVIIATTNMHEHFTPKTMACPGALVRRFTYDYVLAAKKPYIREKTETLN
AJE25834.1/1-2291BRBV          ALEDKGKVFNSPVIIATTNMHEHFTPKTMACPGALVRRFTYDYVLSAKKPYIREKTETLN
QPB41068.1/1-2274BRBV          ALEDKGKVFNSPVIIATTNMHEHFTPKTMACPGALVRRFTYDYILAAKKPYIREKTETLN
YP_009352243.1/1-2218BRAV      ALEDKGTLFTSPVIIATTNLSDAFTPITMACPEALQRRFHFDYNLEAK----WKKGYHLD
AAT01740.1/1-2311FMDV          SLEDKGKPFNSKVIIATTNLYSGFTPRTMVCPDALNRRFHFDIDVSAKDGY--KTNNKLD

OBRV/1-2283                    VRKALAASGEECPCGLFEFDCPLLNGEAIDLTPV-----RDTPTVESVYELIELVYNEVM
UFQ04588.1/1-2283BRBV          VRKALNATGHECPCGLFEFDCPLLNGEAIDLTPV-----RDTPAVESVYELIELVYNEVM
AJE25834.1/1-2291BRBV          VRKALAASGQECPCGLFEFDCPLLNGEAIELTPV-----RNTPTVESVYELIELVYNEVT
QPB41068.1/1-2274BRBV          VRKALAASGHDCPCGLFEFDCPLLNGEALDLTPV-----RETPVVESVYELIELVYNEVM
YP_009352243.1/1-2218BRAV      VKRALQPTGKPAN-ELFEEDYPLLNGQAVMFVAN-----KMCPAIDSAYELIEAVYAAVI
AAT01740.1/1-2311FMDV          IIKALEDT-HTNPVAMFQYDCALLNGMAVEMKRMQQDVFKPQPPLQNVYQLVQEVIDRVE

                                                        3A
OBRV/1-2283                    DRRTVSDLKILKQMGRYAT------LDDVRRRKPVVIPFSGPGYQPGRDINASPEMQE--
UFQ04588.1/1-2283BRBV          DRRTVSDLKILKQMGKCTT------LDNIKRRKPAFTPFSGQGYQPGRDINASPEMQE--
AJE25834.1/1-2291BRBV          DRRTVSDLKVLKQMGKHVT------LDDIRKRKPTVIPFSGQGYQPGRDINASPDMQE--
QPB41068.1/1-2274BRBV          DRRAVSDLRILKQMGKYMT------LDDIKKRKPAVIPFTGRSYQAGRDINASPEMQE--
```

Fig. S4

```
                                                 3A
YP_009352243.1/1-2218BRAV     ERRDVAKVGIVKQV---KT------LHDKLK---ASLP-RGRGYRCDREVNLKTDEAE--
AAT01740.1/1-2311FMDV         LHEKVSSHPIFKQISVPSQKSVLYFLIEKGQHEAAIEFFEGMVHDSIKE-ELRPLLQQTS

OBRV/1-2283                   ---RLLKYLVKH-EHLDAALNFY---------------NEECDEE--------------V
UFQ04588.1/1-2283BRBV         ---KLLKYLVKH-EHLDAALNFY---------------NEECDED--------------I
AJE25834.1/1-2291BRBV         ---KVLKYLVKN-EHLDAALNFY---------------NEECDEE--------------I
QPB41068.1/1-2274BRBV         ---KLLKYLVKN-EHLDAALNFY---------------NEECDED--------------V
YP_009352243.1/1-2218BRAV     ---RMFRYLLNR-DPTLAG-EFL---------------EKECDPE--------------L
AAT01740.1/1-2311FMDV         FVKRAFKRLKENFEVVALCLTLLANIVIMIRETRKRQQNKTLDEAEKNPLETSGASTVGF

OBRV/1-2283                   RAKWGPSIGEYLKVKTLWMKVKK--------YSHLF--LTGLMLIGNMLLLYLNNRTPED
UFQ04588.1/1-2283BRBV         RTKWGPSIGEYLKVKTLWMKVKK--------YSHLF--LTGLMLIGNMLLLYLNNRTPEE
AJE25834.1/1-2291BRBV         RVKWGPSIGEYLKVKTLWMKVKK--------YSHLF--LTGLMLIGNMLLLYLNNKTPEE
QPB41068.1/1-2274BRBV         RDKWGPSIGKYLEVKTLWMKVKK--------YSHLF--LTGLMLIGNMLLLYLNNRTPEE
YP_009352243.1/1-2218BRAV     ADKYLPLLREHTGKSKLWTTLTK--------HCDLF--LHGLLLVANLVTLYFQNRKPR-
AAT01740.1/1-2311FMDV         RERAPPGHKVSDDVNSEPTKPAEEQPQAEGPYAGPLERQKPLKVKAKLPQQEGPYAGPME

                                                     3B                     3C
OBRV/1-2283                   KKKKKKNKTEEDTTAKEGPYGGHAKPP-VKVDKLKVNPLITTESGNPPTDMQMVVLKNTQ
UFQ04588.1/1-2283BRBV         KKKKKKNKAEEESTTKEGPYGGQAKPP-VKVDKLKVNPLITTESGNPPTDMQMVVLKNTQ
AJE25834.1/1-2291BRBV         KKKKKRTKTEEDNTSKEGPYGGQAKPP-VKVDKLKVNPLITTESGNPPTDMQMVVLKNTQ
QPB41068.1/1-2274BRBV         RKKKKKNKTDEDNTTKEGPYGGQAKPP-VKVDKLKVNPLITTESGNPPTDMQMVVLKNTQ
YP_009352243.1/1-2218BRAV     ----------------RQGPYGG--KPTVVKRKTVEAPNLVATESGAPPTDMQQHVLRNVR
AAT01740.1/1-2311FMDV         RQKPLKVKAKA-PVVKEGPYEGPVKKP-VAL-KVKAKNLIVTESGAPPTDLQKMVMGNTK

OBRV/1-2283                   PISLVRDGQVVATCCALGVFGTTYLVPYHLFEESFDTLVIGDRHLKEQDYKLDTFELN--
UFQ04588.1/1-2283BRBV         PISLIRDGQIVATCCALGIFGTTYLVPYHLFEESFDTLVIGDRHLKEQDYKLDTFELK--
AJE25834.1/1-2291BRBV         PISLVRDGQIVATCCALGVFGTTYLVPYHLFEESFDTLVIGDRHLKEQDYKLDTFELR--
QPB41068.1/1-2274BRBV         PISLVRDGQIVATCCALGVFGTTYLVPYHLFEESFDTLVIGDRHLKERDYKLDTFELR--
YP_009352243.1/1-2218BRAV     PISLVCDGKVVSMCCGFGVFGNCYLVPNHMFEETFDTILLGETPLKKKDYEVINLETG--
AAT01740.1/1-2311FMDV         PVELILDGKTVAICCATGVFGTAYLVPRHLFAEKYDKIMLDGRAMTDSDYRVFEFEIKVK

OBRV/1-2283                   -GGKTSDVAALTLNKGARVRDITSHFRDEVKVTKNSPVVGCVKNTMVGQLVFNGTAAGFK
UFQ04588.1/1-2283BRBV         -DGKISDVAALTLNKGARVRDITSHFRDEVKVTKNSPVVGCVKNTTVGQLVFNGTAAGFK
AJE25834.1/1-2291BRBV         -DGKVSDVAALTLNKGARVRDITSHFRDEVKVTKNSPVVGCVKNTTVGQLVFNGTAAGFK
QPB41068.1/1-2274BRBV         -DGKVSDVAAITLNKGARVRDITSHFRDEVKVTRNSPVVGCVKNTTVGQLVFNGTAAGFK
YP_009352243.1/1-2218BRAV     -DG-VSDAALLHVFKGPRVKDMTMHFRDEVRIPKGTTVAGCVNSHEFGRLVFTGTALTFK
AAT01740.1/1-2311FMDV         GQDMLSDAALMVLHRGNRVRDITKHFRDTARMKKGTPVVGVINNADVGRLIFSGEALTYK

OBRV/1-2283                   DHIVCSDGDTLPNMFVYAANTQYGYCGSGILVKDGAHTVVIGIHSAGGNGRGYASCVTRS
UFQ04588.1/1-2283BRBV         DHIICSDGDTLPNMFVYTANTQYGYCGSGILVKDGSHTVIIGIHSAGGNGKGYASCVTRS
AJE25834.1/1-2291BRBV         DHIICSDGDTLPNMFVYTANTQYGYCGSGILVKDGSHTVIIGIHSAGGNGKGYASCVTRS
QPB41068.1/1-2274BRBV         DHIICSDGDTLPNMFVYTANTQYGYCGSGILVKDGSHTVIIGIHSAGGNGKGYASCVTRS
YP_009352243.1/1-2218BRAV     DVIVCSDGDELPNVFAYKAATQRGYCGSPVLVKNSAHTVVVGIHSAGGNGNGYASCVTRS
AAT01740.1/1-2311FMDV         DIVVCMDGDTMPGLFAYRAATKAGYCGGAVLAKDGADTFIVGTHSAGGNGVGYCSCVSRS

                                                 3D
OBRV/1-2283                   ALLALRGREKPE--LEGLMLGQQPGE-RVHVSRKTKLVPTVAYGVFRPEFGPAPLSNSDT
UFQ04588.1/1-2283BRBV         ALLALRGRERPE--LEGLMLGQQPGE-KVHVSRKTKLAPTVAYGVFRPNYGPAALSNSDT
AJE25834.1/1-2291BRBV         ALLALRGRERPE--LEGLMLGEQPGE-KVHVSRKTKLAPTVAYGVFRPNYGPAALSNSDT
QPB41068.1/1-2274BRBV         ALLALRGRERPE--LEGLMLGEQPGE-KVHVSRKTKLAPTVAYGVFRPNYGPAALSNSDT
YP_009352243.1/1-2218BRAV     VLLKVKRMLDPDVHLEGLIVDTREGEERVHVARKSKLYPTLAHAVFKPEFGPAPLSNSDS
AAT01740.1/1-2311FMDV         MLLKMKAHIDPEPHHEGLIVDTRDVEERVHVMRKTKLAPTVAHGVFNPEFGPAALSNKDL

OBRV/1-2283                   RLNEGVVLDDVIFSKHKSNVVLD-NKDLALYRLCAAEYASHLHGVLGKTGAPLTNTEAVL
UFQ04588.1/1-2283BRBV         RLSEGVVLDNVIFSKHNSNVVLD-EKDLALYRLCAAEYASHLHNVLGKTGTPLTNVEAVL
AJE25834.1/1-2291BRBV         RLSEGVVLDNVIFSKHNSNVVLE-EKDLALYRLCAAEYASHLHNVLGKTGAPLTNVEAVL
QPB41068.1/1-2274BRBV         RLNEGVVLDNVIFSKHKSNVVLD-EKDLALYRLCAAEYASHLHNVLGKTGAPLTNVEAVL
YP_009352243.1/1-2218BRAV     RLNPGIVLDNSIFSKHTACVELNYEQSVEFTQACY-DYADKLFGVIGRNNGPLTLFEAVK
AAT01740.1/1-2311FMDV         RLNEGVVLDEVIFSKHKGDAKMS-EEDKALFRRCAADYASRLHSVLGIANAPLSIYEAIK

OBRV/1-2283                   GIEGLDAMEPNTAPGLPWALQHKRRHDLLDFTTGEMQPALAERFQQLEDMDYTFECQTFL
```

Fig. S5

```
UFQ04588.1/1-2283BRBV       GIDGLDAMEPNTAPGLPWALQHKRRHDLLDFTTGEMQPALAERFEQLENMDYSFECQTFL
AJE25834.1/1-2291BRBV       GIDGLDAMEPNTAPGLPWALQHKRRHDLLDFTTGEMQPALAERFEQLENMNYSFECQTFL
QPB41068.1/1-2274BRBV       GIDGLDAMEPNTAPGLPWALQHKRRHDLLDFTTGEMQPALAERFEQLENMDYTFECQTFL
YP_009352243.1/1-2218BRAV   GIEGLDAMETDTGPGLPWSKDNIRRPALIDFEAGTVSETIQKRLDEFEMGRFEFECQTFL
AAT01740.1/1-2311FMDV       GVDGLDAMEPDTAPGLPWALQGKRRGALIDFENGTVGPEVEAALKLMERREYKFACQTFL


OBRV/1-2283                 KDEIRPNEKVRAGKTRIVDVLPLEHIIFSRKYLGRFCAAMHRNYGPNIGSAVGCDPDIAW
UFQ04588.1/1-2283BRBV       KDEIRPNEKVKAGKTRIVDVLPLEHIVFSRKYLGRFCAAMHRNYGPNIGSAVGCDPDVAW
AJE25834.1/1-2291BRBV       KDEIRPSEKVKAGKTRIVDVLPLEHIVFSRKYLGRFCAAMHRNYGPNIGSAVGCDPDVAW
QPB41068.1/1-2274BRBV       KDEIRPNEKVKAGKTRIVDVLPLEHIVFSRKYLGRFCAAMHRNYGPNIGSAVGCDPDVAW
YP_009352243.1/1-2218BRAV   KDELRPNHKIAVGGTRVVDVLPVEHLIFSRIHLGRFCSHMHLNYGLGIGSAVGCNPDVDW
AAT01740.1/1-2311FMDV       KDEIRPMEKVRAGKTRIVDVLPVEHILYTRMMIGRFCAQMHLNNGPQIGSAVGCNPDVDW


OBRV/1-2283                 QEFGTHFSQYKNVWAVDYTAFDSCHSTQLMSVMADEVFSEAFGFDERARYAIKSLCKTVH
UFQ04588.1/1-2283BRBV       QEFGTHFSQYKNVWAIDYTAFDSCHSTQLMSVMADEVFSDAYGFDERARYAVKSLCKTVH
AJE25834.1/1-2291BRBV       QEFGTHFSQFKNVWAIDYTAFDSCHSTQLMSVMADEVFSDAHGFDERARYAVKSLCKTVH
QPB41068.1/1-2274BRBV       QEFGTHFSQYKNVWAIDYTAFDSCHSTQLMSVMADEVFSDAHGFDERARYAVKSLCKTVH
YP_009352243.1/1-2218BRAV   HQFAVQFQPFKNVWAIDYSAFDASHSVDLLSQMIEAVFSDENGFSVHARDVLHSLETTVH
AAT01740.1/1-2311FMDV       QRFGTHFAQYRNVWDVDYSAFDANHCSDAMNIMFEEVFHTDFGFHPNAEWILKTLVNTEH


OBRV/1-2283                 AYEDKRITIDGGLPSGCSATSILNTVLNNIYVLFALRRTYPNTEPGDFTMIAYGDDLVLA
UFQ04588.1/1-2283BRBV       AYEDKRITIDGGLPSGCSATSILNTVLNNIYVMFALKRTYPNTEMGDYSLIAYGDDLVLA
AJE25834.1/1-2291BRBV       AYEDKRITIDGGLPSGCSATSILNTVLNNIYVMFALKRTYPNTEMGDYSLIAYGDDLVLA
QPB41068.1/1-2274BRBV       AYEDKRITIDGGLPSGCSATSILNTVLNNIYVMFALKRTYPNTEMSDYSLIAYGDDLVLA
YP_009352243.1/1-2218BRAV   AYEDKRYTIKGGLPSGCAATSIINTVLNNIYVLYALRREYPWVTPDDYVMVAYGDDVVIA
AAT01740.1/1-2311FMDV       AYENKRITVEGGMPSGCSATSIINTILNNIYVLYALRRHYEGVELDTYTMISYGDDIVVA


OBRV/1-2283                 SDYDYDMNLVKKSFAVLGHTITPEDKSDKGFTLGKTITDVTFLKRAFERDCALGFYKPVM
UFQ04588.1/1-2283BRBV       SDFDYDINLVKKSFAVLGHTITPEDKSDNGFALGKSITDVTFLKRAFERDCVLGFYKPVM
AJE25834.1/1-2291BRBV       SDFDYDINLVKKSFAILGHTITPEDKSDGGFTLGKSITDVTFLKRAFERDCVLGFYKPVM
QPB41068.1/1-2274BRBV       SDLDYDINLVKKSFAVLGHTITPEDKSDNGFTLGKSITDVTFLKRAFERDCVLGLYKPVM
YP_009352243.1/1-2218BRAV   SDHDFDFNKLVARFAELGHKITPEDKSDRGFQLGLKITDVSFLKRQFVFDHYAGFYKPVM
AAT01740.1/1-2311FMDV       SDNDLDFEALKPHFKSLGQTITPADKSDKGFVLGHSITDVTFLKRHFHMDYGTGFYKPVM


OBRV/1-2283                 DSKVLEAILSFARRGTLQEKLASVAGLAFHSGEEEYNRLFAPFAGSFEIPSYRCLRLRWV
UFQ04588.1/1-2283BRBV       NSKVLEAILSFARRGTLQEKLTSVAGLAFHSGEEEYNRLFSPFVGSFEIPSYRCLRLRWV
AJE25834.1/1-2291BRBV       NSKVLEAILSFARRGTLQEKLTSVAGLAFHSGEEEYNRLFSPFAGSFEIPSYRCLRLRWV
QPB41068.1/1-2274BRBV       DSKVLEAILSFARRGTLQEKLTSVAGLAFHSGEEEYNRLFSPFVGSFEIPSYRCLRLRWV
YP_009352243.1/1-2218BRAV   SSRTLEAILSYARKGQLEEKLVSVAGLAFHSGEREFKRLFAPFDGLFEFPSYSALRLRWY
AAT01740.1/1-2311FMDV       ASKTLEAILSFARRGTIQEKLISVAGLAVHSGPDEYRRLFEPFQGLFEIPSHRSLYLRWV


OBRV/1-2283                 HKMSN--
UFQ04588.1/1-2283BRBV       HKMSN--
AJE25834.1/1-2291BRBV       HKMSN--
QPB41068.1/1-2274BRBV       HKMSN--
YP_009352243.1/1-2218BRAV   RAVSQG-
AAT01740.1/1-2311FMDV       NAVCGDA
```
